# Identification of sequences common to more than one therapeutic target to treat complex diseases: simulating the high variance in sequence interactivity evolved to modulate robust phenotypes

**DOI:** 10.1186/s12864-015-1727-6

**Published:** 2015-07-18

**Authors:** Miguel Angel Varela

**Affiliations:** Department of Physiology, Anatomy and Genetics, University of Oxford, South Parks Road, Oxford, OX1 3QX UK

**Keywords:** Antibody, Antisense oligonucleotide, Decoy, Interactivity, Multispecific

## Abstract

**Background:**

Genome-wide association studies show that most human traits and diseases are caused by a combination of environmental and genetic causes, with each one of these having a relatively small effect. In contrast, most therapies based on macromolecules like antibodies, antisense oligonucleotides or peptides focus on a single gene product. On the other hand, complex organisms seem to have a plethora of functional molecules able to bind specifically to multiple genes or genes products based on their sequences but the mechanisms that lead organisms to recruit these multispecific regulators remain unclear.

**Results:**

The mutational biases inferred from the genomic sequences of six organisms show an increase in the variance of sequence interactivity in complex organisms. The high variance in the interactivity of sequences presents an ideal evolutionary substrate to recruit sequence-specific regulators able to target multiple gene products. For example, here it is shown how the 3’UTR can fluctuate between sequences likely to be complementary to other sites in the genome in the search for advantageous interactions. A library of nucleotide- and peptide-based tools was built using a script to search for candidates (e.g. peptides, antigens to raise antibodies or antisense oligonucleotides) to target sequences shared by key pathways in human disorders, such as cancer and immune diseases. This resource will be accessible to the community at www.wikisequences.org.

**Conclusions:**

This study describes and encourages the adoption of the same multitarget strategy (e.g., miRNAs, Hsp90) that has evolved in organisms to modify complex traits to treat diseases with robust pathological phenotypes. The increase in the variance of sequence interactivity detected in the human and mouse genomes when compared with less complex organisms could have expedited the evolution of regulators able to interact to multiple gene products and modulate robust phenotypes. The identification of sequences common to more than one therapeutic target carried out in this study could facilitate the design of new multispecific methods able to modify simultaneously key pathways to treat complex diseases.

**Electronic supplementary material:**

The online version of this article (doi:10.1186/s12864-015-1727-6) contains supplementary material, which is available to authorized users.

## Background

Genome-wide association studies have established that most human traits and diseases are caused by a combination of environmental and genetic causes. The vast majority of these causes have relatively small effects on a particular trait. For example, intelligence is highly heritable, but the variability of the trait corresponds to the additive nature of multiple genotypes and their interactions [1]. Potentially, very different allelic combinations could produce equally gifted individuals. Equally, many human diseases, such as cancer, or immune disorders, should also fall into this polygenic category [2]. In contrast, many therapeutic approaches focus on modulating the abundance of a single gene product or targeting a single receptor. Developing a single compound to target the gene that has the largest effect in most patients can be a good strategy in some cases. Nevertheless, even when the effect on the phenotype is sufficient, with time, the success of treatment can be hampered by the upregulation of pathways other than the one that is being targeted.

Redundant pathways are very common in complex organisms, and some have been maintained over millions of years [3]. During evolution, higher organisms have recruited multitarget regulatory elements that are capable of modifying these robust networks and reconcile maintaining functionality under mutational pressure with being able to rapidly adapt the phenotype to the environment. Of note among these regulatory elements are microRNAs (miRNA) and heat shock protein 90 (Hsp90). miRNAs are short RNA molecules (19–24 nucleotides long)[4, 5] that generally downregulate gene expression by guiding the RNA-induced silencing complex (RISC) to a number of complementary mRNAs. In fact, miRNAs have been shown to play a role in increasing the canalization and tunability of networks [6–8]. In contrast, Hsp90 stabilizes proteins after synthesis and exposure to heat. Hsp90 has been described as an evolutionary capacitor that allows genomes to store genetic diversity in complex traits without exposing them to natural selection [9, 10].

Recently, the development of therapies that modulate these multi-regulatory elements has been actively pursued, e.g., therapies modulating miRNA levels in cancer [11, 12] or in cardiovascular diseases [13, 14]. Additionally, the inhibition of Hsp90 activity has also shown potential in multiple studies, mainly in cancer research [15–17]. The main caveat of modulating natural combinatory elements is that they can potentially target many mRNAs or proteins that are unrelated to the particular pathology of interest, causing off-target effects. Therefore, it would be ideal to design tailored multi-regulatory tools.

The aim of this work was to study the strategy by which organisms modify complex traits in order to inspire the design of new therapeutic tools that are able to alter robust disease phenotypes. First, we analyzed on the features of complex genomes that maintain high variances in the interactivity of their sequences and mutational biases, facilitating the search for advantageous interactions with other sequences. Then, a library was built of molecular effectors to potentially target multiple key gene products involved in human disorders that display complex pathological networks.

## Methods

### DNA and amino acid datasets

DNA and protein sequences were accessed from the National Center for Biotechnology Information (NCBI) database (www.ncbi.nlm.nih.gov) and Ensemble (www.ensembl.org) for the following species: Ec (*Escherichia coli* CFT073 assembly eschColi_536), At (*Arabidopsis thaliana* assembly TAIR9), Ce (*Caenorhabditis elegans* assembly WBcel235), Dm (*Drosophila melanogaster* assembly BDGP5), Mm (*Mus musculus* C57BL/6 J assembly *GRCm38/mm10*) and Hs (*Homo sapiens* assembly GRCh37). Sequences were identified and subjected to BLAST searches using tools found on the Ensemble (www.ensembl.org), UCSC Genome Browser (www.genome.ucsc.edu) and Galaxy websites (www.galaxyproject.org) [18]. BLAST searches in cDNA were performed on a random collection of 48 sequences with different percentages of low- or high-frequency dinucleotides using the following parameters, search sensitivity: exact matches; E: 100; filter: none; w: 2; wink: 1. Ratios of dinucleotide frequencies were calculated using total genomic counts.

Sequences present in more than one gene product involved in human diseases and not included in any other mRNA or protein were searched from a collection of 308 gene and 1105 peptide sequences related to cancer, and 72 gene and 344 peptide sequences related to immune disorders. These sequences were subjected to searches using custom scripts written in visual basic. Rather than searching for conserved protein domains and functional sites using alignment algorithms, the search was performed by dividing each cDNA or protein of interest into a matrix of multiple cells of 12 nucleotides or 4 amino acids, respectively, and then, a script was run to search for repeated cells. This method increases the sensitivity of the search by removing the negative influence of non-common flanking sequences. Based on these sequences, a library was built containing nucleotide- and peptide-based tools capable of simultaneously targeting key cancer pathways or other diseases with a strong genetic background. This resource is accessible to the community at www.wikisequences.org. This webpage will also be open to users to upload their own sequences of interest and for linking to their manuscripts. Protein topology was assigned according to *Uniprot* annotation (www.uniprot.org) and TMHMM (http://www.cbs.dtu.dk/services/TMHMM/), whereas peptide structure was predicted using PEP-FOLD, which is based on hidden Markov models [19, 20].

### Statistical analysis

Statistical analyses were conducted in R (http://www.r-project.org/). Plots of the sequence frequency in the cDNA relative to the length and percentage of high-frequency dinucleotides (CA, AT, GC, AG) were constructed using local spline fitting (‘locfit’ function) to generate a smoothed surface. Error bars represent 95 % confidence intervals (binomial distributions) in comparison with random expectations.

## Results

### Sequences as targets of multispecific molecular effectors

**Fig. 1:**
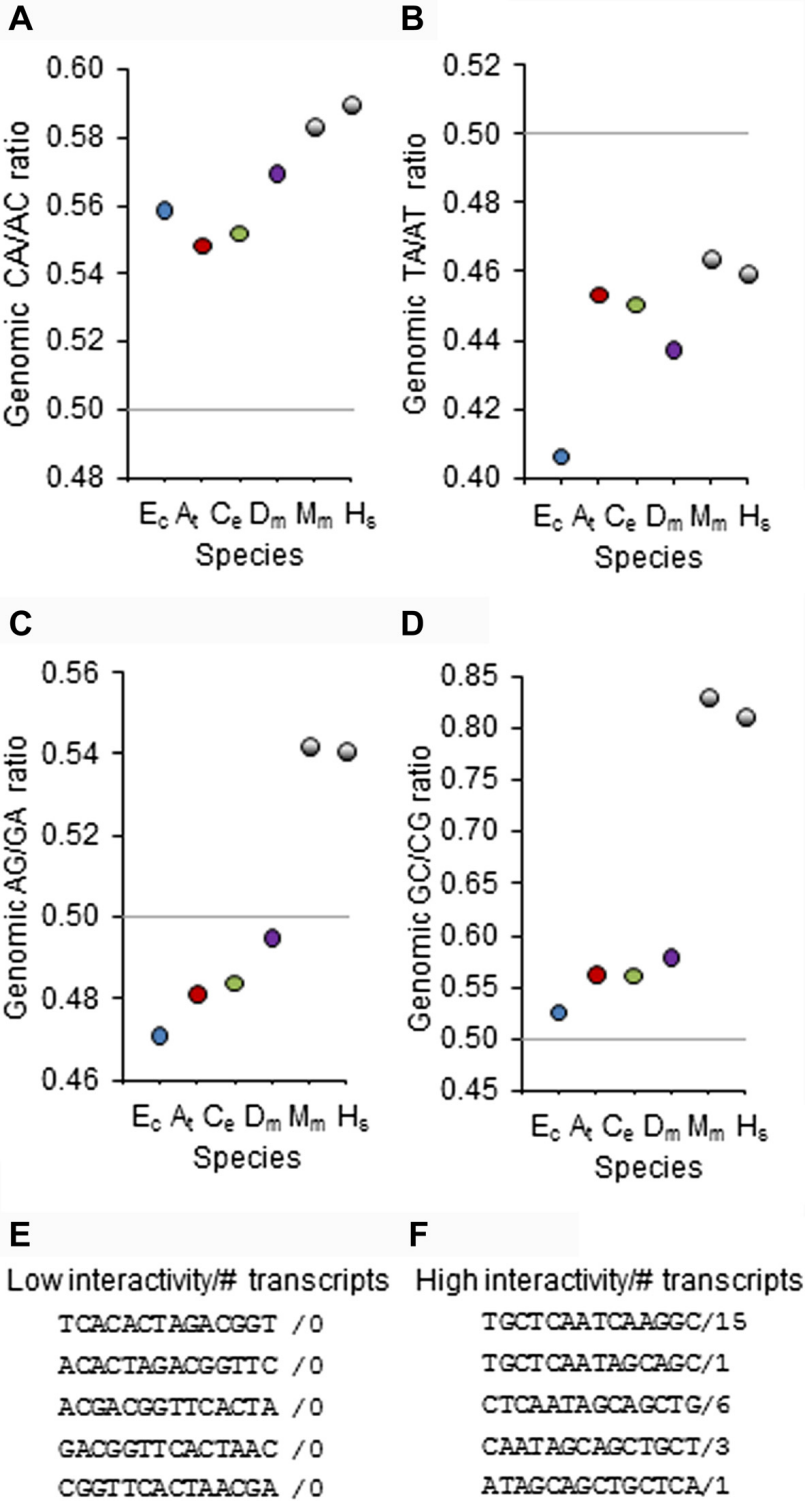
Bias of dinucleotide frequencies observed in species of different complexity. Mutational biases CA/AC (**a**), AT/TA (**b**), AG/GA (**c**), GC/CG (**d**). Examples of sequences and their frequencies in human cDNA comprising a random collection of low-frequency (**e**) or high-frequency dinucleotides are shown (**f**). Horizontal lines represent ratio 1:1, i.e., no bias. Error bars are contained in data points and represent 95 % confidence intervals (binomial distributions) in comparison with random expectations. Ec (*Escherichia coli* CFT073), At (*Arabidopsis thaliana*), Ce (*Caenorhabditis elegans*), Dm (*Drosophila melanogaster*), Mm (*Mus musculus*), Hs (*Homo sapiens*)

Many interactions between genes involve sequence-specific regulation of promoters, transcripts or proteins [21, 22]. The nucleotide distribution in the sequences of organisms of different complexity is important for understanding how the different layers of regulation are recruited. In Fig. 1, we can observe changes in the dinucleotide frequencies observed in species of different complexity: Ec (*Escherichia coli* CFT073), At (*Arabidopsis thaliana*), Ce (*Caenorhabditis elegans*), Dm (*Drosophila melanogaster*), Mm (*Mus musculus*) and Hs (*Homo sapiens*). Some of these changes could reflect mutational biases that were revealed after the release of the pressure to bias codon composition to optimize translation proficiency. Others could correspond to new mutational biases. One such bias is the well-known high mutability and scarcity of CpGs in vertebrates [23]. Interestingly, also note the decrease in the TA/AT ratio from the lowest values, found in *E. coli*, to those in eukaryotes (TA, not AT, is present in two of three stop codons).

**Fig. 2:**
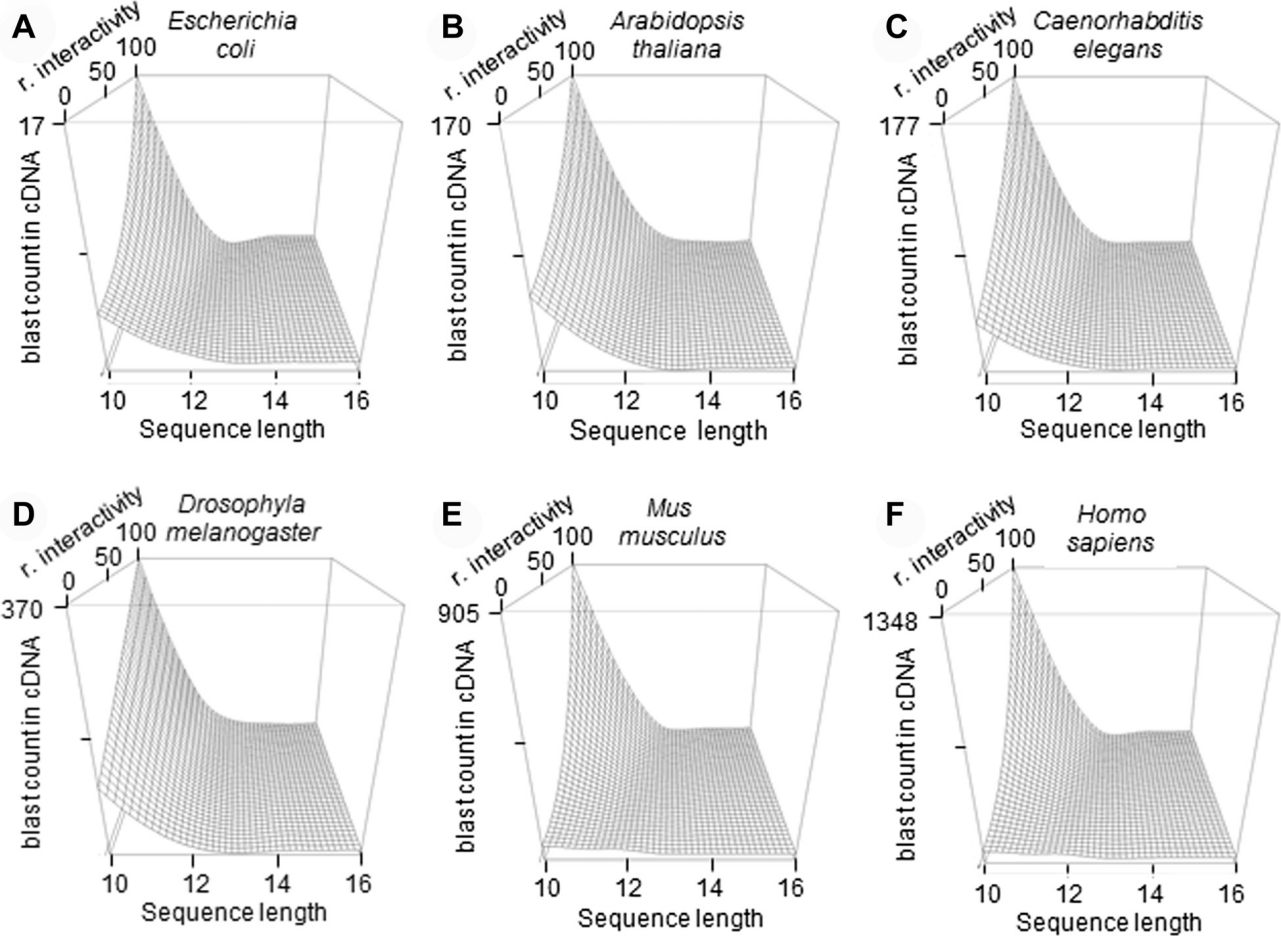
Increase in the variance of sequence interactivity in the human and mouse genomes. 3D plots of sequence frequency in cDNA relative to the length and relative interactivity (r. interactivity) measured as the percentage of high-frequency (CA, AT, GC, AG) vs low-frequency dinucleotides (AC, TA, CG, GA) show an increase in the variance of sequence interactivity in *Mus musculus* and *Homo sapiens *(**e** and **f**) in comparison with *Escherichia coli* (**a**), *Arabidopsis thaliana*(**b**), *Caenorhabditis elegans*(**c**) and *Drosophyla melanogaster*(**d**)

Although Fig. 1 provides a good summary of how the nucleotide distribution varies with organism complexity, it provides substantially less information about how these asymmetries can affect the frequency distribution of longer sequences. Consequently, plots for the frequency of 10–16 nt sequences were constructed for each species by blasting sequences with different proportions of the most common dinucleotides against the cDNA of each species (Fig. 2). We can observe that these dinucleotide asymmetries can add up to striking increases in the frequency of longer sequences. Figure 2 also reveals how *Mus musculus* and *Homo sapiens* show an increased slope in sequence frequency depending on the nucleotide composition when compared with other organisms. Considering complementarity as the basis for interaction with regulatory elements, we could also describe this observation as an increase in the variance of sequence interactivity.

**Fig. 3:**
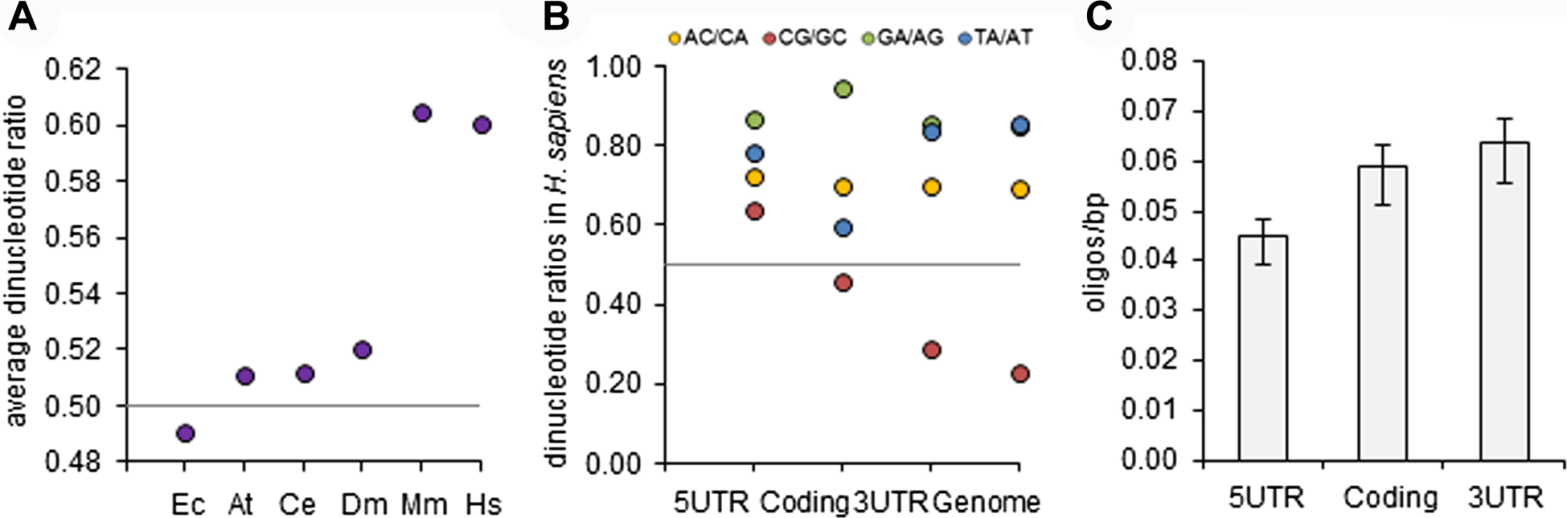
High variance in the interactivity of sequences facilitates the recruitment of multispecific regulators. (**a**) Average ratio of dinucleotide frequencies with the same base composition observed in species of different complexities. (**b**) Human sequences in the 3’UTR have a very similar nucleotide composition to the rest of the genome in comparison with the 5’UTR, increasing the likelihood of interactions with other sequences by complementarity. (**c**) Relative proportion per nucleotide of the common nucleotide sequences targeting genes of therapeutic interest identified in this work (considering the average nucleotide size of the 5’UTR, coding sequence and 3’UTR as 200, 1340 and 800 bp, respectively). Error bars are contained in data points in (A) and (B) and represent 95 % confidence intervals (binomial distributions) in comparison with random expectations. Horizontal lines represent ratio 1:1, i.e., no bias. Ec (*Escherichia coli* CFT073), At (*Arabidopsis thaliana*), Ce (*Caenorhabditis elegans*), Dm (*Drosophila melanogaster*), Mm (*Mus musculus*), Hs (*Homo sapiens*)

During the evolution of complexity, organisms have allowed or recruited mutational biases that have shaped the frequency of some of these sequences to the point of having combinations of nucleotides or amino acids that are rarely formed, whereas others act as attractors regardless of any direct selective pressure. For example, the frequencies of sequences in human cDNA comprising a random collection of low-frequency or high-frequency dinucleotides in human cDNA are shown in Fig. 1 (E and F, respectively). In Fig. 3a, it is shown how these asymmetries in the ratios of dinucleotide frequencies are especially evident in mouse and in human when compared with less complex species. In human genes the strength of the asymmetries increases gradually from 5’ to 3’ and is of similar strength in the 3’UTR as in the rest of the genome (Fig. 3b). Human sequences in the 3’UTR have a very similar dinucleotide composition to the rest of the genome in comparison with the 5’UTR, increasing the likelihood of interactions with other sequences by complementarity. The high variance in the interactivity of sequences could facilitate the adoption of sequence-specific regulators able to target multiple gene products. 3’UTR is also the main target of miRNAs and, interestingly, the distribution of the nucleotide sequences described in this study as having therapeutic potential for multitargeting also show a significant trend toward being more commonly located in the 3’UTR, with decreasing frequency approaching the 5’UTR (Fig. 3c).

### Building a library of nucleotide- and peptide-based multispecific macromolecules

Multispecific molecular effectors that target sequences common to more than one gene or receptor have the potential to alter robust disease phenotypes. The identification of short sequences common to multiple genes by alignment algorithms can be hampered by the negative influence of non-common flanking sequences. Using a script that increases the sensitivity of the search by removing this influence, a library of nucleotide- and peptide-based tools was built to contain more than one gene product involved in a particular human disease that is not included in any other mRNA or protein. An extract of this library is presented here in Tables 1 and 2 (and in Supplementary Information Additional file 1: Table S1 and Additional file 2: Table S2). This library will also be available at the website www.wikisequences.org, which is open to the community for uploading other sequences of interest and linking them to manuscripts.

**Table 1:** Antisense gapmer oligonucleotides that exclusively demonstrate reverse complementarity to multiple cDNAs related to particular disorders. All nucleotides are linked by phosphorothioate linkages *, and conformationally restricted nucleotide monomers, such as tricycle-DNA, LNAs and MOEs, are preceded by +. Additional sequences can be found in Table S1 and will be updated at www.wikisequences.org

Antisense oligonucleotides	Targets
	Cancer
+T*+T*+G*A*T*G*G*G*G*A*A*C*T*+T*+G*+G	*ABL1,BLK,FGR,HCK.*
+G*+C*+C*A*A*G*C*C*A*A*A*+G*+T*+C	*ACVR2B,ARAF,CHECK1,FGR.*
+A*+G*+G*T*C*C*A*G*T*T*T*+C*+T*+G	*ATR,MKI67,PNK3.*
+T*+G*+T*C*A*G*C*T*G*T*C*+A*+T*+T	*BCL2,HIF2,PSMA6.*
+T*+T*+G*G*T*T*T*C*C*T*T*+T*+G*+C	*BIRC5,HK2,USP34.*
+G*+G*+C*C*A*G*G*C*C*A*A*A*+G*+T*+C	*CAMK2A,CDK4,CDK16,FIP1L1,*
	*HCK,IRAK1,PDGFRA.*
+A*+C*+C*A*G*C*T*G*C*T*T*G*+A*+A*+G	*CCNE2,FGR1,FGR3,FGR4.*
+G*+G*+C*C*A*G*G*C*C*A*A*A*G*+T*+C*+A	*CDK4,CDK16,FIP1L1,HCK,MAPK(1&9).*
+G*+C*+C*A*T*C*C*A*C*T*T*+C*+A*+C	*C-KIT,C-MET,CSF1R,FGFR1,FGFR3,*
	*FGFR4,FIP1L1,MST1R,PDGFRA.*
+T*+T*+G*C*G*G*G*C*A*G*C*C*+A*+G*+G	*CSK,EPHB2,FGR3,FGR4,TNK2,RIPK4.*
+G*+T*+T*A*C*A*A*G*C*A*T*+C*+A*+T	*DOCK7,TGFB2,USP14.*
+A*+G*+C*C*A*C*T*G*G*A*T*+G*+T*+G	*E2F1,FGR1,FGR3,FGR4,HSPA12A.*
+T*+G*+T*G*A*T*A*C*T*T*T*+C*+T*+G	*EBAG9,MCL1,PTGS2,TMED7-TICAM2.*
+A*+C*+A*T*C*A*C*T*C*T*G*G*T*G*+G*+G*+T	*EGFR,FGFR1,HER2.*
+C*+A*+C*C*T*G*G*T*A*G*G*C*+G*+C*+A	*FGFR1,FGFR4,TERT.*
+T*+C*+A*C*T*G*T*A*C*A*C*+C*+T*+T	*FGR1,FGR3,GRB7.*
+C*+A*+C*C*T*G*G*T*A*G*G*C*+G*+C*+A	*FGR1,FGR4,TERT.*
	Immunological diseases
+C*+C*+A*A*C*C*T*T*C*A*+C*+A*+C	*CARD9,IL7R,RPL5.*
+T*+C*+T*C*C*T*T*C*C*T*C*T*G*+C*+T*+T	*KRT17,NLRP1.*
+C*+C*+G*T*G*G*G*T*C*C*C*T*G*+G*+C*+A	*ILR6,TLR9.*

**Table 2:** Peptide sequences present in multiple proteins involved in particular human diseases. These peptides could be used as decoys, antigens to raise antibodies, or if aiming to intracellular targets delivered directly in the format of stapled peptides, incorporated as loops into naturally occurring cyclic peptides, or used after conjugation with molecules that aid in cellular uptake. Additional sequences can be found in Table S2 will be updated at www.wikisequences.org

Sequence	Targets
	Cancer
ILLLDEATSALDTESE.	*ABCB1* ^*e*^&^*i*^ *,ABCB4* ^*i*^ *,ABCB11* ^*i*^ *.*
KVLGSGAFGTVYKG.	*EGFR* ^*i*^ *,ERBB2* ^*i*^ *,ERBB4* ^*i*^ *.*
KVAVKMLKS.	*CSF1R* ^*i*^ *,FGFR1* ^*i*^ *,PDGFRB* ^*i*^
VHRDLAARNVLV.	*CSK* ^*i*^ *,EGFR* ^*e*^ *,EPHA8* ^*i*^ *,ERBB2* ^*e*^ *,*
	*ERBB4* ^*e*^ *,FLT3* ^*e*^ *,JAK1* ^*i*^ *,SRMS* ^*i*^ *.*
RIY**THQSDVWS**YGVT	*ERBB3* ^*e*^ *,EGFR* ^*e*^ *.*
VWELMTFG.	
YQLYSRTSGKH.	*FGF8* ^e^ *,FGF17* ^e^ *,FGF18* ^e^ *.*
PSQRPTFKQLVEDLDR.	*FGFR1* ^*i*^ *,FGFR2* ^*i*^ *,FGFR3* ^e^ *.*
ERSPHRPILQAGLPAN.	*FGFR1* ^e^ *,FGFR2* ^e^ *,FGFR3* ^e^ *,FGFR4* ^*e*^ *.*
MEKKLHAVPA.	*FGFR1* ^*e*^ *,FGFR4* ^*e*^ *.*
SEMEMMKMIGKHKNII	*FGFR1* ^*i*^ *,FGFR2* ^*i*^ *,FGFR3* ^*i*^ *.*
NLLGACTQ.	
**THQSDVWS**F.	*FGFR1* ^i^ *,FGFR2* ^i^ *,FGFR3* ^e^ *,FGFR4* ^*e*^ *.*
KCIHRDLAARNVLVT	*FGFR1* ^*i*^ *,FGFR3* ^e^ *,FGFR4* ^i^ *.*
EDNVMKIADFGLAR.	
FSVLYTVPAT.	*FZD1* ^*t*^ *,FZD2* ^*t*^ *,FZD4* ^*t*^ *,FZD7* ^*t*^ *,*
	*FZD10* ^*t*^ *.*
YPERPIIFLS.	*FZD1* ^i^ *,FZD2* ^e^ *,FZD4* ^*t*^ *,FZD5* ^e^ *,FZD7* ^e^ *,*
	*FZD8* ^i^ *,FZD9* ^*t*^ *,FZD10* ^i^ *.*
FLALDLGGTNFRVL.	*HK1* ^i^ *,HK2* ^i^ *,HK3* ^i^ *,HKDC1* ^i^.
QLELPVKYA.	*ITGAm* ^*e*^ *,ITGAD* ^*e*^ *,ITGAx* ^*e*^ *.*
LLCDKVQKDDIEVRF.	*REL* ^i^ *,NFKB1* ^i^ *,NFKB2* ^i^ *.*
	*Immunological diseases*
LCLEERDWLPG.	*TLR7* ^i^ *,TLR9* ^i^ *.*
GLFWANLRAAIN.	*TRL1* ^i^ *,TRL6* ^i^ *,TRL10* ^i^ *.*
IEKSYKSIFVL.	*TRL1* ^i^ *,TRL6* ^i^ *,TRL10* ^i^ *.*

^e^extracellular, ^i^internal, ^t^transmembrane site, bold: repeated motif

These sequences could be targeted alone or in combination, depending on the pathological pathways that are involved. For therapeutic purposes, these sequences could be manipulated using small-interfering RNAs (siRNAs), antisense oligonucleotides such as locked nucleic acids (LNAs) [24, 25], tricyclo-DNA [26, 27], 2'-O-methoxyethyls (MOEs) [28, 29], morpholinos [30], peptide nucleic acids (PNAs) [31] or other silencing effectors expressed in viral constructs or plasmids [32] (Table 1).

Incorporating conformationally restricted nucleotide monomers into oligonucleotides through the use of intercalators or extra bridges between atoms, as in the case of LNAs or tricyclo-DNA, increases the resistance to nucleases and *tm*, allowing for short oligonucleotides to retain much of their activity *in vitro*. Furthermore, several studies have shown that it is possible to improve the potency of antisense oligonucleotides *in vivo* by reducing their length to less than 17-mer [27, 33, 34]. The high variance in sequence interactivity of complex organisms shown in this study facilitates the presence of targets of this size range common to more than one mRNA or protein related to a particular trait or disease.

Although the sequences that are shown in Table 1 are in the format of antisense gapmer oligonucleotides, by aiming at DNA targets, these sequences could alternatively be used for transcriptional gene silencing or targeted gene modification with zinc-finger nucleases (ZFNs), transcription activator-like effector nucleases (TALENs) or clustered regulatory interspaced short palindromic repeat (CRISPR)/Cas-based methods [35]. Some of these sequences could also be used in the design of RNA-binding proteins, either alone [36, 37] or in combination with RNA cleavage domains [38]. Reductions in the size of the targets or the introduction of abasic sites, e.g., by using spacers, could further increase the number of genes of interest that are targeted, but it could also affect the expression of other genes; thus, further analysis would be necessary.

**Fig. 4:**
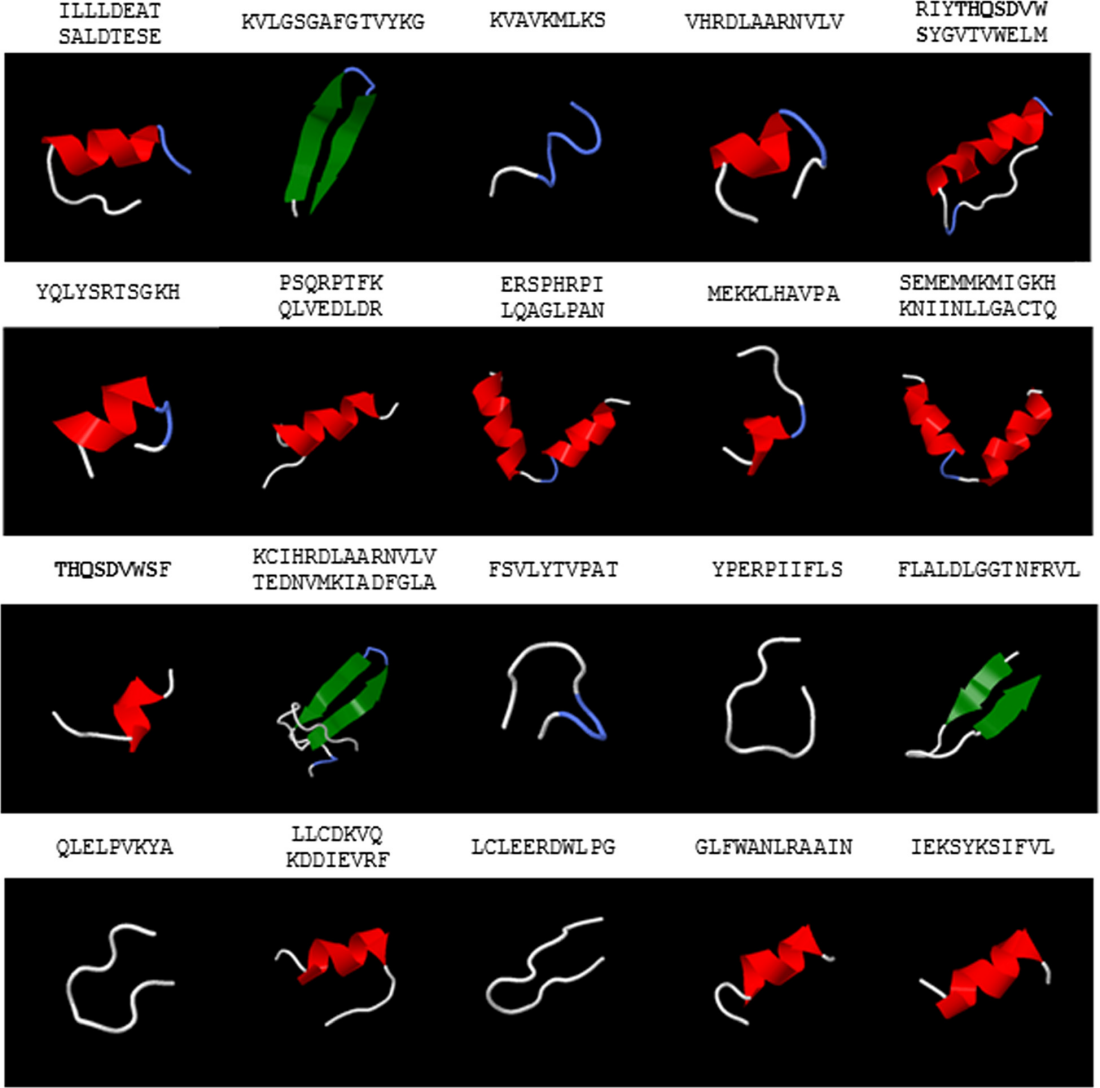
Prediction of peptide structures. Further characterization of the peptides included in Table 2, red: helical, green: extended, blue: coil. Peptide structure was predicted using PEP-FOLD, which is based on hidden Markov models. The structure of these peptides is of importance to define their usefulness depending on their application (eg to raise antibodies, as decoys etc.)

In regards to peptide sequences, targets present in multiple proteins and involved in particular human diseases could be employed as decoys [39, 40], to raise antibodies [41], delivered directly in the form of stapled peptides [42–44] or incorporated as loops into naturally occurring cyclic peptides [45, 46] (Table 2). The structure predicted for these peptides can be observed in Fig. 4. Finally, sequences present in multiple homing peptides could specifically recognize more than one type of tumor [47, 48] (Additional file 3: Table S3). The accession numbers of all the peptide and nucleotide sequences that were searched for targets shared by key pathways can be found in the (Additional files 4: Table S4) and (Additional file 5: Table S5). Further details on how the search was performed are in (Additional file 6: Figure S1).

## Discussion

This study describes and encourages the adoption of the strategy that is used in organisms to modify complex traits to treat diseases with robust pathological phenotypes. Comparative genomics reveals a striking increase in the frequency variance of sequences depending on nucleotide composition in complex organisms. The high variance in the interactivity of sequences presents an ideal evolutionary substrate to recruit sequence-specific regulators that target multiple gene products. Coding sequences retain their functionality, while other sequences, mainly the 3’UTR, fluctuate between nucleotide compositions that are very likely to be complementary to other sequences in the genome in the search for advantageous interactions. This feature of the human genome has facilitated the recruitment of an ever increasing sophistication in the simultaneous regulation of multiple gene products. Recent advances in biotechnology will allow us to continue forming the next layer of regulation, but this time, the tools will be assembled in laboratories and stored online.

### Sequences as targets of multispecific molecular effectors

The interface between the genetic information contained in DNA sequences and the environment is shaped by a sophisticated network of regulatory elements. This interface in complex organisms can be understood from not only the perspective of positive selection but also other non-adaptative mechanisms shaping the mutational biases that tend to form these sequences, rather than natural selection acting on each sequence individually. The result of this process is that some sequences are formed more frequently, regardless of any direct selective pressure.

Natural selection can initially fixate enzymes or miRNAs with few targets, and then, more targets are added to the pool by the evolutionary inertness of the sequences, with natural selection only acting to remove the most deleterious of the interactions. Although outside the scope of this study, maintaining a high variance in the interactivity of its elements and the interplay of genetic drift and multitarget regulation could be key to complex organisms outcompeting others by their pace of innovation. An example of the increase in sequence interactivity associated with complexity can be observed in the way in which miRNAs operate in plants and animals. In plants, perfect complementarity of miRNAs to their target mRNAs is required to regulate gene expression. In contrast, in animals, miRNA complementarity is only necessary for the 5' bases 2 through 7 of the miRNA, and as a result, the same miRNA can target many different mRNAs [49, 50].

In complexity race scenarios, organisms recruit mutational biases and tend to frequently show mutations toward states that have previously been favorable for building complexity. The more organized a system is, the more sophisticated are the mechanisms that bias the way in which tensions are released by mutations or the way in which these mutations are corrected or expressed to increase complexity. We are in a unique moment in that soon (at least from an evolutionary point of view), we will have the technology to not only ameliorate complex human diseases but also to modify human traits by deliberately modulating the expression of chosen combinations of genes.

### Building a library of nucleotide- and peptide-based multispecific macromolecules

Modulating gene expression using multitarget regulatory elements could be the key for ameliorating robust disease phenotypes. The best example in this regard is most likely cancer, a disease for which the perception is shifting from a gene-centered, uncontrolled growth scenario to another perspective based on highly regulated developmental networks [51]. However, the situation should be similar for many other human disorders in which severity has been shown to be determined by more than one gene and/or network. Recently, combinatory approaches have been actively pursued, e.g., developing therapies targeting the regulation of miRNA levels. A number of such therapies modulating miRNAs [52] or Hsp90 [53] are being studied in clinical trials.

The main disadvantage of modulating naturally occurring multi-regulatory mechanisms is the off-target effects that could be potentially caused by targeting mRNAs or proteins that are unrelated to the targeted pathology. For example, a particular miRNA can target hundreds of genes, and while it may be very valuable in developing an antitumoral therapy, it has not evolved to become the perfect switch for a particular kind of cancer. There is room for improvement by modifying the sequence of miRNAs or finding other sequences common to mRNAs or proteins of interest.

The advantages of tailored multitarget tools could be, first, to circumvent the regulation of parallel pathways and elemental redundancy in networks. To increase patient coverage and avoid the appearance of resistance mechanisms in cancer treatments, it seems logical to develop therapies that are able to target multiple gene products or regulators. Second, using a single sequence would reduce the cost of production because synthesis, quality control and toxicology would be limited to one molecule, or a few in the case of using a combinatorial approach. An additional advantage of utilizing a multitarget approach would be to take advantage of any synergistic effect that could be derived from gene epistasis. Epistasis is often overlooked merely because of the difficulty of using genetic interactions to estimate heritability accurately [54, 55]. Lastly, from the point of view of therapeutic efficacy, it is preferable to use short sequences that are common to several genes of interest to improve their delivery *in vivo*, as large sizes can limit biodistribution.

Some of the applications of these designs could be to carry cancer drugs to tumors, modulate the immune system or block the action of a series of gene products. For example, a possible design could consist of an antibody directed to the common peptide sequences present in the extracellular domain of the fibroblast growth factor receptors (FGFRs) or their ligands (Table 2 and Fig. 4). Theses receptors play an important role in cell proliferation and survival [56] and they are the target of a number of therapeutic molecules, such as FP-1039, a soluble receptor that acts as a decoy for all the mitogenic FGF ligands [57]. Furthermore, there is already a growing interest in using bispecific or multispecific antibodies [58] whether the targeting is achieved by modularity [59–61] or even by developing antibodies able to bind to more than one epitope in a single antigene recognition surface [62–64]. The peptide structure shown in Fig. 4 was predicted using PEP-FOLD, which is based on hidden Markov models [19, 20]. The structure of these peptides is of importance to define their usefulness depending on their application (eg to raise antibodies or as decoys) [65]. For instance, alpha-helical peptides have hydrophobic and hydrophilic faces that can interact with the membrane to being internalized.

Ideally, a combination of targets would be available for each different genetic background leading to the disease, and the corresponding cocktail of macromolecules would be validated as being able to ameliorate the pathology. In reality, generating an ideal set of guidelines for the gene therapy of complex diseases is not a simple task of linking mutant genotypes with the tools that are able to modify the expression of a number of genes. Despite recent advances, predicting phenotypes from genotypes is not straightforward [66]. Additionally, only some of the common sequences would be amenable for the design of therapeutic tools. Therefore, a future set of guidelines for the use of these macromolecules will presumably require multiple rounds of bioinformatic analysis and empirical validation. In this context, a database such as wikisequences.org containing these and other sequences and open to comments and performance reports across different studies could be valuable to facilitate empirical validation.

## Conclusions

Most human traits and diseases are caused by multiple environmental and genetic causes, with each one of them having a relatively small effect. In contrast, most macromolecular therapies focus on targeting a single receptor or modulating the abundance of a single gene product. This work defines and promotes the adoption of the same multitarget strategy that has evolved in organisms to modify complex traits in order to simultaneously target key pathways of human disorders with robust phenotypes, such as cancer and immune disorders.

The genomic sequences of mouse and human show an increase in the frequency variance of sequences depending on the nucleotide composition in comparison with organisms of lower complexity. The mutational biases inferred from the genomic sequences can lead to a high variance in the interactivity of the sequences, facilitating the recruitment of sequence-specific regulators that are able to target multiple gene products. For example, sequences in the 3’UTR seem to be more free of constraints in comparison with the 5’UTR to mutate towards a very similar nucleotide composition to the rest of the genome, increasing the likelihood of interacting with other sequences in the genome due to complementarity.

Taking into account the importance of multitargeting for modifying robust phenotypes, a script to search for sequences with the potential for multitarget regulation and genes of interest in the medical literature was utilized to build a library of nucleotide- and peptide-based tools to target more than one gene product involved in a particular human disease that is not included in any other mRNA or protein. This resource is open to other users at www.wikisequences.org to upload other designs of interest, comment on their performance and link them to other manuscripts.

Among the main advantages of tailored multitarget macromolecules is the limitation of the off-target effects caused by modulating natural multi-regulatory elements such as miRNAs and Hsp90. Second, multitargeting circumvents the regulation of parallel pathways and developing redundancy in cancer networks. Third, multitargeting would reduce the cost of production because a smaller number of different molecules would be needed to target the same number of gene products or receptors. Fourth, combinatorial approaches can take advantage of any synergistic effect that could be derived from gene epistasis. Finally, from the point of view of delivery, it is preferable to use short sequences that are common to several genes of interest rather than compound designs that would hamper *in vivo* efficacy. This last point of delivery is always cited as the limiting factor for many avant-garde therapies based on macromolecules, but advances not only in delivery but also in multitargeting could be also important for macromolecules to overcome the current advantages of small molecules in the clinic.

## Additional files

Additional file 1: Table S1. Antisense gapmer oligonucleotides that exclusively demonstrate reverse complementarity to multiple cDNAs related to particular disorders. Additional file 2: Table S2. Peptide sequences present in multiple proteins involved in particular human diseases. Additional file 3: Table S3. Peptide sequences present in multiple homing peptides that specifically recognize cancer cells or tumor vessels. Additional file 4: Table S4. Accession numbers of nucleic acid sequences that were searched for targets shared by key pathways cancer and immune diseases to facilitate the design of new multispecific methods to treat complex diseases. Additional file 5: Table S5. Accession numbers of peptides that were searched for targets shared by key pathways cancer and immune diseases to facilitate the design of new multispecific methods to treat complex diseases. Additional file 6: Figure S1. Identification of sequences shared by multiple genes to form a library of nucleotide- and peptide-based tools capable of simultaneously targeting key pathways.
