# Supplementary material for: Identification of sequences common to more than one therapeutic target to treat complex diseases: simulating the high variance in sequence interactivity evolved to modulate robust phenotypes
Source: BMC Genomics. 2015 Jul 18;16(1):530. doi: 10.1186/s12864-015-1727-6 (PMC4506634; doi:10.1186/s12864-015-1727-6)
Supplement: Additional file 1: Table S1. — Antisense gapmer oligonucleotides that exclusively demonstrate reverse complementarity to multiple cDNAs related to particular disorders. [file 12864_2015_1727_MOESM1_ESM.docx]

**Table S1 Antisense gapmer oligonucleotides that exclusively demonstrate reverse complementarity to multiple cDNAs related to particular disorders.** The sequences are shown in the format of antisense gapmer oligonucleotides, with all nucleotides being linked by phosphorothioate linkages *, and conformationally restricted nucleotide monomers, such as tricycle-DNA, LNAs and MOEs, are preceded by +. Alternatively, these sequences could be employed for targeted gene modification, transcriptional gene silencing, or correspond to reverse complementary targets of RNA-binding proteins. Additional sequences will be updated at [www.wikisequences.org](http://www.wikisequences.org).

| Antisense oligonucleotides | Targets |
| --- | --- |
|  | Cancer |
| +A*+G*+A*G*C*C*A*C*C*T*G*A*+A*+C*+C | *ABCB1 ERBB3.* |
| +A*+G*+C*C*C*C*C*A*C*C*A*G*T*C*+C*+A*+C | *AKT1 AKT2 PKN3.* |
| +A*+C*+C*A*C*G*T*T*C*T*T*C*T*C*+C*+G*+A | *AKT1 VEGRF2.* |
| +A*+G*+C*T*C*T*T*A*T*A*A*+G*+T*+C | *AR CTNNB1.* |
| +T*+G*+T*C*A*T*T*C*A*C*A*C*+C*+A*+G | *BIRC2 BIRC7 PARP1.* |
| +C*+A*+T*G*T*T*C*T*A*C*C*+C*+A*+T | *BIRC2 MDM2.* |
| +T*+G*+C*A*A*A*T*A*G*G*G*+C*+A*+T | *BIRC2 MDM4.* |
| +T*+C*+T*G*C*C*A*G*G*A*C*+G*+C*+T | *BIRC5 FOXM1.* |
| +A*+T*+A*A*C*T*G*T*T*G*T*+T*+C*+T | *BIRC6 BRAF.* |
| +G*+T*+T*T*A*G*T*T*G*C*A*+G*+C*+A | *BIRC6 CTNNB1.* |
| +A*+C*+C*T*C*G*A*T*G*T*T*+G*+G*+G | *BIRC6 DMT1.* |
| +A*+G*+A*C*A*T*A*C*T*A*A*+T*+T*+C | *BIRC6 EIF4E.* |
| +G*+C*+T*T*G*T*T*C*T*A*C*C*A*+G*+G*+A | *BIRC6 MDM2.* |
| +T*+T*+C*C*T*G*A*A*G*G*A*+C*+A*+T | *BIRC6 TEP1.* |
| +T*+A*+C*A*G*A*A*C*A*A*T*T*+C*+C*+A | *BRAF MKI67.* |
| +C*+C*+C*A*C*A*C*T*T*G*C*+C*+T*+G | *CCDND1 HER2.* |
| +T*+T*+G*G*T*T*C*G*G*+C*+A*+G | *CCND1 C-MET.* |
| +T*+C*+C*A*G*T*G*G*G*G*T*+C*+C*+T | *CCND2 HK2.* |
| +G*+A*+C*C*C*T*C*T*T*G*G*C*A*+G*+C*+A | *C-MYC HIF2.* |
| +A*+T*+G*T*A*A*T*C*A*T*A*C*+A*+T*+T | *CTLA4 IGF1.* |
| +T*+T*+T*A*G*A*G*A*A*G*A*+T*+G*+C | *EIF4E PNK3.* |
| +T*+T*+T*T*G*T*G*C*A*C*C*+A*+A*+C | *EPCAM MDM2.* |
| +G*+G*+A*T*G*G*G*C*C*G*G*T*+G*+A*+G | *FGR1 FGFR2 PRKA.* |
| +T*+G*+T*A*T*T*G*T*A*C*A*T*+A*+A*+T | *HK2 BCL2.* |
| +T*+T*+T*C*A*T*T*G*C*A*T*G*+A*+A*+G | *KRAS MDM2.* |
| +T*+T*+A*T*G*G*T*C*T*T*C*+A*+A*+G | *MCL1 MTDH.* |
| +G*+C*+T*G*T*T*C*T*G*C*C*+T*+C*+A | *MKI67 STAT3.* |
| +G*+T*+G*G*G*C*G*A*T*G*G*+T*+G*+A | *MRP1 MRP2.* |
| +T*+T*+C*A*T*G*T*C*C*A*T*+G*+T*+A | *PDGFRA SCFR.* |
| +A*+C*+C*T*C*C*T*T*G*G*C*G*T*+A*+G*+T | *RRBP1 TGFB1. TFGB2.* |
| +G*+G*+C*C*C*C*T*G*A*T*T*A*+T*+A*+C | *SIRT1 VEGRF2.* |
| +A*+T*+A*A*T*G*G*A*A*T*T*T*+G*+G*+G | *UCHL5 VEGRF2.* |
| +G*+C*+T*G*G*C*A*T*C*A*T*A*+A*+G*+G | *VEGRF1 VEGRF2.* |
