# Supplementary material for: Identification of sequences common to more than one therapeutic target to treat complex diseases: simulating the high variance in sequence interactivity evolved to modulate robust phenotypes
Source: BMC Genomics. 2015 Jul 18;16(1):530. doi: 10.1186/s12864-015-1727-6 (PMC4506634; doi:10.1186/s12864-015-1727-6)
Supplement: Additional file 2: Table S2. — Peptide sequences present in multiple proteins involved in particular human diseases. [file 12864_2015_1727_MOESM2_ESM.docx]

**Table S2 Peptide sequences present in multiple proteins involved in particular human diseases.** These sequences could be used as decoys, antigens to raise antibodies, or if aiming to intracellular targets delivered directly in the format of stapled peptides, incorporated as loops into naturally occurring cyclic peptides, or used after conjugation with molecules that aid in cellular uptake. Some of the applications of these designs could include blocking or mimicking the action of a series of gene products, delivering cancer drugs to tumors or modulating the immune system. Additional sequences will be updated at [www.wikisequences.org](http://www.wikisequences.org).

| Sequence | Targets |
| --- | --- |
|  | Cancer |
| DFGLATV. | *ARAF1 BRAF CHECK1 DCLK1 DCLK2 MAPK3 RAF1.* |
| SDVWSFG. | *AXL CSK EPHA10 EPHB6 FGR FER FES FGFR1 FGFR2 FGFR3 FRK FYN HCK* |
|  | *ITK LCK LYN MAP3K10 MAP3K11 MATK MET MST1R NTRK1 SRMS PTK* |
|  | *SRC SYK TEC TIE1 TXK TYK2 YES1.* |
| LARAGF. | *BIRC2 BIRC8 XIAP.* |
| VAVKML. | *BRAF CSF1R DDR2 FGFR1 FGFR2 FGFR3 FGFR4 FIP1L1 FLT1 FLT3 FLT4 KDR* |
|  | *KIT PDGFRA MUSK PDGFRB RET TYRO3.* |
| KIGDFG. | *BRAF EIF2AK2 LTK MAPK4 RAF1 TNK2 TYK2.* |
| HCCLNP.* | *CCR4 CCR6 CX3CR1 CXCR3 CXCR4 CXCR5.* |
| LRRVGD. | *CLEC2L MCL1 TERT.* |
| QSDVWSYG. | *CSF1R EGFR ERBB2 ERBB3 ERBB4.* |
| SDVWSFGV. | *CSK EGFR EPHB1 EPHB2 EPHB3 EPHA4 EPHA5 EPHA6 EPHA7 EPHA8 ERBB2* |
|  | *ERBB4 FIP1L1 MET MST1R PDGFRA PDGFRB RET SRMS SYK ZAP70.* |
| VISNIN.* | *EIF2B5 EPHA3.* |
| VLENNYTA*. | *FGF8 FGF17 FGF18.* |
| VETDTFGS*. | *FGF8 FGF17 FGF18.* |
| PVQTSQ.* | *FOXM1 TNFRSF8.* |
| LTWFLAAG. | *FZD1 FZD2 FZD4 FZD5 FZD8 FZD9 FZD10.* |
| MPNLLGHT.* | *FZD1 FZD2 FZD9.* |
| LRFFLCS.* | *FZD2 FZD7 FZD5 FZD9 FZD10.* |
| EEEKKTK.* | *IGFR1 CNGA3.* |
| SNSIYPW.* | *ITGA1 ITGA2 ITGA10 ITGA11 HOXB7.* |
| QIGSYFGS.* | *ITGA1 ITGA2 ITGA10 ITGA11 ITGAD ITGAE ITGAX.* |
| LLVGAPM.* | *ITGA1 ITGA2 ITGA4 ITGA9 ITGA11 ITGA2B.* |
| DLLVGAPL.* | *ITGA5 ITGAB.* |
| PFEKNCG.* | *ITGAD ITGAL ITGAM ITGAX.* |
| YKVGFFKR.* | *ITGAL ITGAx.* |
| VGRTGAGKSSLT. | *MRP1 MRP2.* |
| LFSGSLRMNLDPF. | *MRP1 MRP2.* |
| RLAQEA. | *RHPN1 TNFRSF8.* |
| HTLLDALE. | *TNFRSF10A TNFRSF10b.* |
|  | Immunological diseases |
| IIQACRG.* | *CASP1 CASP3 CASP6 CASP14.* |
| VLGAPRYQ.* | *ITGAM ITGAD ITGAX.* |
| LEHLDLS.* | *LRIG3 TLR2 TLR4 TLR10.* |
| WSDIEC.* | *PPARGC1A TNFRSF10A TNFRSF10B.* |
| GSVRGGDE. | *REL NFKB2.* |
| DGIFET.* | *TNC CEP350.* |
| ILILLEPI. | *TRL1 TRL2 TRL6 TRL10.* |
| HLDLSFN. | *TLR1 TLR6 PIDD.* |
| LEILDVS. | *TLR2 TLR5 TLR6.* |
| LTRLDL. | *TRL5 TRL8 PIDD.* |
| SKKTVFV. | *TLR7 TLR8.* |

*extracellular site.
