# Supplementary material for: Identification of sequences common to more than one therapeutic target to treat complex diseases: simulating the high variance in sequence interactivity evolved to modulate robust phenotypes
Source: BMC Genomics. 2015 Jul 18;16(1):530. doi: 10.1186/s12864-015-1727-6 (PMC4506634; doi:10.1186/s12864-015-1727-6)
Supplement: Additional file 3: Table S3. — Peptide sequences present in multiple homing peptides that specifically recognize cancer cells or tumor vessels. [file 12864_2015_1727_MOESM3_ESM.docx]

**Table S3 Peptide sequences present in multiple homing peptides that specifically recognize cancer cells or tumor vessels.** Additional sequences will be periodically updated at [www.wikisequences.org](http://www.wikisequences.org).

| Sequence | Target tumor | References |
| --- | --- | --- |
| ALRDRPM. | colorectal cancer | [1] |
|  | colon cancer | Us7452965b2 |
| CDCRGDCFC. | tumor neovasculature | [2] |
|  | hepatic and pancreatic cancer | [3] |
|  | breast cancer | [4] |
|  | melanoma/breast cancer* | [5] |
| CEYQLDVE. | pancreatic cancer | [6] |
|  | pancreatic cancer | [7] |
| CGKRK. | epidermal squamous cell cancers | [8] |
|  | breast cancer | [9] |
| CGQKRTRGC. | melanoma/breast cancer* | [9] |
|  | melanoma/breast cancer* | [10] |
| CGLIIQKNEC. | prostate cancer | [11] |
|  | multiple tumors | [12] |
| CGNSNPKSC. | gastric cancer | [13] |
|  | gastric cancer | [14] |
| CNAGESS. | multiple tumors | [12] |
|  | multiple tumors | Us20090214429a1 |
| CNRRTKAGC. | esophagus and gastroesophageal cancer | [14] |
|  | cervical cancer | [15] |
| CRSRKG. | pancreatic cancer | [6] |
|  | pancreatic cancer | [7] |
| CVLNGRMEC. | melanoma/breast cancer* | [16] |
|  | melanoma | [4] |
| ESSKNC. | multiple tumors | Us20090214429a1 |
| EPAYQRFL. | melanoma/breast cancer* | [17] |
|  | neuroblastoma/melanoma/breast cancer* | [18] |
|  | neuroblastoma/melanoma/breast cancer* | [19] |
| EPKPKKAPAKK. | breast cancer/fibrosarcoma | [20] |
|  | breast cancer | [9] |
| GCNGRC. | hepatic and pancreatic cancer | [3] |
|  | melanoma/breast cancer* | [16] |
|  | melanoma/breast cancer* | [21] |
|  | multiple tumors | [22] |
|  | multiple tumors | [12] |
|  | melanoma/breast cancer* | Us6180084b1 |
|  | melanoma/breast cancer* | Us6180084b1 |
| IFLLWQR. | multiple tumors | [23] |
|  | multiple tumors | [12] |
|  | metastasis | Us7470658b2 |
| KDEPQRRSAR | breast tumor | [24] |
| KGCGTRQCW. | melanoma | Us6180084b1 |
|  | kaposi's sarcoma | Us6180084b1 |
| KGVSLSYR. | breast cancer | [25] |
|  | breast cancer | [26] |
| LSAKPAPPKP | peritoneal carcinomatosis | [27] |
| PEKFRPM. | colorectal cancer | [1] |
|  | colon cancer | Us7452965b2 |
| SMSIASP. | gastric cancer, liver metastasis | [28] |
|  | gastric cancer | [29] |
| SRESPHP. | medullary thyroid carcinoma | [30] |
|  | medullary thyroid carcinoma | [31] |
| RGDfK. | ovarian carcinoma | [32] |
|  | glioblastoma, prostate cancer | [33] |
| WGTGLC. | melanoma | Us6180084b1 |
|  | melanoma | Us6180084b1 |

*****see Ross et al. and Rae et al.[34] for a discussion on the identity of MDA-MB-435 cells.

**References**

1. Kelly KA, Jones DA: **Isolation of a colon tumor specific binding peptide using phage display selection**. *Neoplasia N Y N* 2003, **5**:437–444.

2. Dickerson EB, Akhtar N, Steinberg H, Wang Z-Y, Lindstrom MJ, Padilla ML, Auerbach R, Helfand SC: **Enhancement of the antiangiogenic activity of interleukin-12 by peptide targeted delivery of the cytokine to alphavbeta3 integrin**. *Mol Cancer Res MCR* 2004, **2**:663–673.

3. Aoki Y, Hosaka S, Kawa S, Kiyosawa K: **Potential tumor-targeting peptide vector of histidylated oligolysine conjugated to a tumor-homing RGD motif**. *Cancer Gene Ther* 2001, **8**:783–787.

4. Arap W, Haedicke W, Bernasconi M, Kain R, Rajotte D, Krajewski S, Ellerby HM, Bredesen DE, Pasqualini R, Ruoslahti E: **Targeting the prostate for destruction through a vascular address**. *Proc Natl Acad Sci U S A* 2002, **99**:1527–1531.

5. Pasqualini R, Koivunen E, Ruoslahti E: **Alpha v integrins as receptors for tumor targeting by circulating ligands**. *Nat Biotechnol* 1997, **15**:542–546.

6. Joyce JA, Laakkonen P, Bernasconi M, Bergers G, Ruoslahti E, Hanahan D: **Stage-specific vascular markers revealed by phage display in a mouse model of pancreatic islet tumorigenesis**. *Cancer Cell* 2003, **4**:393–403.

7. Rafii S, Avecilla ST, Jin DK: **Tumor vasculature address book: identification of stage-specific tumor vessel zip codes by phage display**. *Cancer Cell* 2003, **4**:331–333.

8. Hoffman JA, Giraudo E, Singh M, Zhang L, Inoue M, Porkka K, Hanahan D, Ruoslahti E: **Progressive vascular changes in a transgenic mouse model of squamous cell carcinoma**. *Cancer Cell* 2003, **4**:383–391.

9. Numata K, Mieszawska-Czajkowska AJ, Kvenvold LA, Kaplan DL: **Silk-based nanocomplexes with tumor-homing peptides for tumor-specific gene delivery**. *Macromol Biosci* 2012, **12**:75–82.

10. Karmali PP, Kotamraju VR, Kastantin M, Black M, Missirlis D, Tirrell M, Ruoslahti E: **Targeting of albumin-embedded paclitaxel nanoparticles to tumors**. *Nanomedicine Nanotechnol Biol Med* 2009, **5**:73–82.

11. Tan M, Burden-Gulley SM, Li W, Wu X, Lindner D, Brady-Kalnay SM, Gulani V, Lu Z-R: **MR molecular imaging of prostate cancer with a peptide-targeted contrast agent in a mouse orthotopic prostate cancer model**. *Pharm Res* 2012, **29**:953–960.

12. Pilch J, Brown DM, Komatsu M, Järvinen TAH, Yang M, Peters D, Hoffman RM, Ruoslahti E: **Peptides selected for binding to clotted plasma accumulate in tumor stroma and wounds**. *Proc Natl Acad Sci U S A* 2006, **103**:2800–2804.

13. Chen J, Wang G, Lu C, Guo X, Hong W, Kang J, Wang J: **Synergetic Cooperation of microRNAs with Transcription Factors in iPS Cell Generation**. *PLoS ONE* 2012, **7**:e40849.

14. Zhi M, Wu K, Hao Z, Guo C, Yao J: **Screening of specific binding peptide targeting blood vessel of human esophageal cancer in vivo in mice**. *Chin Med J (Engl)* 2011, **124**:581–585.

15. Zhang L, Giraudo E, Hoffman JA, Hanahan D, Ruoslahti E: **Lymphatic zip codes in premalignant lesions and tumors**. *Cancer Res* 2006, **66**:5696–5706.

16. Pasqualini R, Koivunen E, Kain R, Lahdenranta J, Sakamoto M, Stryhn A, Ashmun RA, Shapiro LH, Arap W, Ruoslahti E: **Aminopeptidase N is a receptor for tumor-homing peptides and a target for inhibiting angiogenesis**. *Cancer Res* 2000, **60**:722–727.

17. Ahmed S, Mathews AS, Byeon N, Lavasanifar A, Kaur K: **Peptide arrays for screening cancer specific peptides**. *Anal Chem* 2010, **82**:7533–7541.

18. Zhang J, Spring H, Schwab M: **Neuroblastoma tumor cell-binding peptides identified through random peptide phage display**. *Cancer Lett* 2001, **171**:153–164.

19. Askoxylakis V, Zitzmann S, Mier W, Graham K, Krämer S, von Wegner F, Fink RHA, Schwab M, Eisenhut M, Haberkorn U: **Preclinical evaluation of the breast cancer cell-binding peptide, p160**. *Clin Cancer Res Off J Am Assoc Cancer Res* 2005, **11**:6705–6712.

20. Park J-H, von Maltzahn G, Zhang L, Derfus AM, Simberg D, Harris TJ, Ruoslahti E, Bhatia SN, Sailor MJ: **Systematic surface engineering of magnetic nanoworms for in vivo tumor targeting**. *Small Weinh Bergstr Ger* 2009, **5**:694–700.

21. Arap W, Pasqualini R, Ruoslahti E: **Cancer treatment by targeted drug delivery to tumor vasculature in a mouse model**. *Science* 1998, **279**:377–380.

22. Curnis F, Gasparri A, Sacchi A, Cattaneo A, Magni F, Corti A: **Targeted delivery of IFNgamma to tumor vessels uncouples antitumor from counterregulatory mechanisms**. *Cancer Res* 2005, **65**:2906–2913.

23. Hatakeyama S, Sugihara K, Shibata TK, Nakayama J, Akama TO, Tamura N, Wong S-M, Bobkov AA, Takano Y, Ohyama C, Fukuda M, Fukuda MN: **Targeted drug delivery to tumor vasculature by a carbohydrate mimetic peptide**. *Proc Natl Acad Sci U S A* 2011, **108**:19587–19592.

24. Bhojani MS, Ranga R, Luker GD, Rehemtulla A, Ross BD, Van Dort ME: **Synthesis and investigation of a radioiodinated F3 peptide analog as a SPECT tumor imaging radioligand**. *PloS One* 2011, **6**:e22418.

25. Hassan S, Buchanan M, Jahan K, Aguilar-Mahecha A, Gaboury L, Muller WJ, Alsawafi Y, Mourskaia AA, Siegel PM, Salvucci O, Basik M: **CXCR4 peptide antagonist inhibits primary breast tumor growth, metastasis and enhances the efficacy of anti-VEGF treatment or docetaxel in a transgenic mouse model**. *Int J Cancer J Int Cancer* 2011, **129**:225–232.

26. Huang EH, Singh B, Cristofanilli M, Gelovani J, Wei C, Vincent L, Cook KR, Lucci A: **A CXCR4 antagonist CTCE-9908 inhibits primary tumor growth and metastasis of breast cancer**. *J Surg Res* 2009, **155**:231–236.

27. Drecoll E, Gaertner FC, Miederer M, Blechert B, Vallon M, Müller JM, Alke A, Seidl C, Bruchertseifer F, Morgenstern A, Senekowitsch-Schmidtke R, Essler M: **Treatment of peritoneal carcinomatosis by targeted delivery of the radio-labeled tumor homing peptide bi-DTPA-[F3]2 into the nucleus of tumor cells**. *PloS One* 2009, **4**:e5715.

28. Hu S, Guo X, Xie H, Du Y, Pan Y, Shi Y, Wang J, Hong L, Han S, Zhang D, Huang D, Zhang K, Bai F, Jiang H, Zhai H, Nie Y, Wu K, Fan D: **Phage display selection of peptides that inhibit metastasis ability of gastric cancer cells with high liver-metastatic potential**. *Biochem Biophys Res Commun* 2006, **341**:964–972.

29. Bai F, Liang J, Wang J, Shi Y, Zhang K, Liang S, Hong L, Zhai H, Lu Y, Han Y, Yin F, Wu K, Fan D: **Inhibitory effects of a specific phage-displayed peptide on high peritoneal metastasis of gastric cancer**. *J Mol Med Berl Ger* 2007, **85**:169–180.

30. Böckmann M, Drosten M, Pützer BM: **Discovery of targeting peptides for selective therapy of medullary thyroid carcinoma**. *J Gene Med* 2005, **7**:179–188.

31. Böckmann M, Hilken G, Schmidt A, Cranston AN, Tannapfel A, Drosten M, Frilling A, Ponder BAJ, Pützer BM: **Novel SRESPHP peptide mediates specific binding to primary medullary thyroid carcinoma after systemic injection**. *Hum Gene Ther* 2005, **16**:1267–1275.

32. Janssen HLA, Reesink HW, Lawitz EJ, Zeuzem S, Rodriguez-Torres M, Patel K, van der Meer AJ, Patick AK, Chen A, Zhou Y, Persson R, King BD, Kauppinen S, Levin AA, Hodges MR: **Treatment of HCV Infection by Targeting MicroRNA**. *N Engl J Med* 2013, **368**:1685–1694.

33. Ito T, Inoue M, Akamatsu K, Kusaka E, Tanabe K, Nishimoto S: **αvβ3-Integrin-targeting lanthanide complex: synthesis and evaluation as a tumor-homing luminescent probe**. *Bioorg Med Chem Lett* 2011, **21**:3515–3518.

34. Rae JM, Creighton CJ, Meck JM, Haddad BR, Johnson MD: **MDA-MB-435 cells are derived from M14 melanoma cells--a loss for breast cancer, but a boon for melanoma research**. *Breast Cancer Res Treat* 2007, **104**:13–19.
