# Supplementary material for: Identification of sequences common to more than one therapeutic target to treat complex diseases: simulating the high variance in sequence interactivity evolved to modulate robust phenotypes
Source: BMC Genomics. 2015 Jul 18;16(1):530. doi: 10.1186/s12864-015-1727-6 (PMC4506634; doi:10.1186/s12864-015-1727-6)
Supplement: Additional file 4: Table S4. — Accession numbers of nucleic acid sequences that were searched for targets shared by key pathways cancer and immune diseases to facilitate the design of new multispecific methods to treat complex diseases. [file 12864_2015_1727_MOESM4_ESM.docx]

**Table S4 Accession numbers of nucleic acid sequences that were searched for targets shared by key pathways cancer and immune diseases to facilitate the design of new multispecific methods and treat complex diseases.**

| Analyzed genes related to cancer | |  |  |  |  |
| --- | --- | --- | --- | --- | --- |
| ENSG00000000938 | ENSG00000099282 | ENSG00000113721 | ENSG00000135333 | ENSG00000157404 | ENSG00000176105 |
| ENSG00000005471 | ENSG00000099968 | ENSG00000114268 | ENSG00000135404 | ENSG00000157764 | ENSG00000176890 |
| ENSG00000005844 | ENSG00000100030 | ENSG00000114279 | ENSG00000135424 | ENSG00000158106 | ENSG00000177283 |
| ENSG00000005884 | ENSG00000100644 | ENSG00000114638 | ENSG00000135446 | ENSG00000158815 | ENSG00000178568 |
| ENSG00000005961 | ENSG00000100902 | ENSG00000114739 | ENSG00000135605 | ENSG00000159216 | ENSG00000180340 |
| ENSG00000007264 | ENSG00000101017 | ENSG00000115085 | ENSG00000135679 | ENSG00000159399 | ENSG00000182511 |
| ENSG00000010278 | ENSG00000101197 | ENSG00000115232 | ENSG00000136573 | ENSG00000160447 | ENSG00000182578 |
| ENSG00000010810 | ENSG00000101336 | ENSG00000115414 | ENSG00000136997 | ENSG00000160654 | ENSG00000182580 |
| ENSG00000011105 | ENSG00000101412 | ENSG00000115464 | ENSG00000137193 | ENSG00000160683 | ENSG00000182866 |
| ENSG00000012061 | ENSG00000101557 | ENSG00000115738 | ENSG00000137809 | ENSG00000160867 | ENSG00000183317 |
| ENSG00000017427 | ENSG00000101966 | ENSG00000115760 | ENSG00000137875 | ENSG00000160883 | ENSG00000183421 |
| ENSG00000019991 | ENSG00000102225 | ENSG00000116016 | ENSG00000138160 | ENSG00000161638 | ENSG00000183813 |
| ENSG00000023839 | ENSG00000102466 | ENSG00000116106 | ENSG00000138685 | ENSG00000162344 | ENSG00000184216 |
| ENSG00000030304 | ENSG00000102755 | ENSG00000116641 | ENSG00000139292 | ENSG00000162434 | ENSG00000184937 |
| ENSG00000037280 | ENSG00000102854 | ENSG00000116750 | ENSG00000140285 | ENSG00000162733 | ENSG00000185499 |
| ENSG00000041982 | ENSG00000102882 | ENSG00000117472 | ENSG00000140379 | ENSG00000162924 | ENSG00000186810 |
| ENSG00000044524 | ENSG00000103222 | ENSG00000117650 | ENSG00000140443 | ENSG00000163098 | ENSG00000186895 |
| ENSG00000044574 | ENSG00000103653 | ENSG00000118777 | ENSG00000140678 | ENSG00000163251 | ENSG00000187098 |
| ENSG00000050748 | ENSG00000104290 | ENSG00000118971 | ENSG00000141639 | ENSG00000163599 | ENSG00000188389 |
| ENSG00000055332 | ENSG00000104689 | ENSG00000118972 | ENSG00000141736 | ENSG00000164078 | ENSG00000188763 |
| ENSG00000061938 | ENSG00000104894 | ENSG00000119888 | ENSG00000141738 | ENSG00000164171 | ENSG00000196468 |
| ENSG00000062524 | ENSG00000105221 | ENSG00000120659 | ENSG00000141867 | ENSG00000164362 | ENSG00000197122 |
| ENSG00000065361 | ENSG00000105329 | ENSG00000120885 | ENSG00000142208 | ENSG00000164611 | ENSG00000198355 |
| ENSG00000066056 | ENSG00000105397 | ENSG00000120889 | ENSG00000143119 | ENSG00000164930 | ENSG00000198400 |
| ENSG00000066468 | ENSG00000105550 | ENSG00000120949 | ENSG00000143127 | ENSG00000165025 | ENSG00000198625 |
| ENSG00000068078 | ENSG00000105668 | ENSG00000121380 | ENSG00000143384 | ENSG00000165304 | ENSG00000198793 |
| ENSG00000070193 | ENSG00000105976 | ENSG00000121594 | ENSG00000143799 | ENSG00000165731 | ENSG00000204389 |
| ENSG00000070388 | ENSG00000106025 | ENSG00000121966 | ENSG00000144191 | ENSG00000165868 | ENSG00000204390 |
| ENSG00000070808 | ENSG00000106123 | ENSG00000122025 | ENSG00000144668 | ENSG00000166589 | ENSG00000206383 |
| ENSG00000070886 | ENSG00000106211 | ENSG00000125508 | ENSG00000145191 | ENSG00000166598 | ENSG00000213281 |
| ENSG00000073734 | ENSG00000106366 | ENSG00000125844 | ENSG00000145216 | ENSG00000166851 | ENSG00000213949 |
| ENSG00000073756 | ENSG00000106462 | ENSG00000125968 | ENSG00000145242 | ENSG00000167325 | ENSG00000215328 |
| ENSG00000074966 | ENSG00000106537 | ENSG00000126453 | ENSG00000145604 | ENSG00000167601 | ENSG00000225830 |
| ENSG00000075388 | ENSG00000107165 | ENSG00000126602 | ENSG00000146648 | ENSG00000168036 | ENSG00000226704 |
| ENSG00000077150 | ENSG00000107831 | ENSG00000126803 | ENSG00000147257 | ENSG00000168329 | ENSG00000231738 |
| ENSG00000077782 | ENSG00000108219 | ENSG00000127324 | ENSG00000147649 | ENSG00000168610 | ENSG00000234258 |
| ENSG00000078061 | ENSG00000108846 | ENSG00000128052 | ENSG00000147654 | ENSG00000168785 | ENSG00000234475 |
| ENSG00000078098 | ENSG00000109320 | ENSG00000128917 | ENSG00000148773 | ENSG00000169083 | ENSG00000235941 |
| ENSG00000078579 | ENSG00000109971 | ENSG00000129473 | ENSG00000149489 | ENSG00000169896 | ENSG00000236251 |
| ENSG00000080224 | ENSG00000110092 | ENSG00000129566 | ENSG00000149554 | ENSG00000170390 | ENSG00000236279 |
| ENSG00000083457 | ENSG00000110330 | ENSG00000130167 | ENSG00000151247 | ENSG00000170606 | ENSG00000237724 |
| ENSG00000085117 | ENSG00000110651 | ENSG00000130758 | ENSG00000151422 | ENSG00000171094 | ENSG00000243414 |
| ENSG00000085563 | ENSG00000110911 | ENSG00000130816 | ENSG00000154229 | ENSG00000171552 | ENSG00000254087 |
| ENSG00000089685 | ENSG00000111206 | ENSG00000132155 | ENSG00000154928 | ENSG00000171791 | ENSG00000260027 |
| ENSG00000091409 | ENSG00000111432 | ENSG00000132646 | ENSG00000155366 | ENSG00000171848 | ENSG00000276536 |
| ENSG00000091879 | ENSG00000111728 | ENSG00000133083 | ENSG00000155760 | ENSG00000173110 | ENSG00000276582 |
| ENSG00000092445 | ENSG00000111816 | ENSG00000133216 | ENSG00000156427 | ENSG00000173327 | ENSG00000276977 |
| ENSG00000092969 | ENSG00000112486 | ENSG00000133703 | ENSG00000156510 | ENSG00000174059 | ENSG00000278183 |
| ENSG00000096384 | ENSG00000112619 | ENSG00000134198 | ENSG00000156515 | ENSG00000174775 |  |
| ENSG00000096717 | ENSG00000113013 | ENSG00000134259 | ENSG00000156738 | ENSG00000174804 |  |
| ENSG00000096968 | ENSG00000113263 | ENSG00000134853 | ENSG00000156886 | ENSG00000175054 |  |
| ENSG00000097007 | ENSG00000113578 | ENSG00000134970 | ENSG00000157240 | ENSG00000175305 |  |
| Analyzed genes related to immunological diseases | | |  |  |  |
| ENSG00000067182 | ENSG00000105426 | ENSG00000135837 | ENSG00000143369 | ENSG00000169194 | ENSG00000198793 |
| ENSG00000073756 | ENSG00000107758 | ENSG00000136244 | ENSG00000145777 | ENSG00000169896 | ENSG00000204490 |
| ENSG00000077150 | ENSG00000109471 | ENSG00000136689 | ENSG00000156886 | ENSG00000170581 | ENSG00000206439 |
| ENSG00000077238 | ENSG00000109819 | ENSG00000136869 | ENSG00000160712 | ENSG00000174123 | ENSG00000223952 |
| ENSG00000085563 | ENSG00000111537 | ENSG00000137462 | ENSG00000162594 | ENSG00000174125 | ENSG00000228321 |
| ENSG00000091181 | ENSG00000113302 | ENSG00000137752 | ENSG00000162924 | ENSG00000174130 | ENSG00000228849 |
| ENSG00000091592 | ENSG00000120889 | ENSG00000138794 | ENSG00000163464 | ENSG00000180871 | ENSG00000228978 |
| ENSG00000095303 | ENSG00000120910 | ENSG00000138814 | ENSG00000164047 | ENSG00000185104 | ENSG00000230108 |
| ENSG00000100385 | ENSG00000122406 | ENSG00000139263 | ENSG00000164305 | ENSG00000185291 | ENSG00000232810 |
| ENSG00000101916 | ENSG00000125538 | ENSG00000140678 | ENSG00000167754 | ENSG00000187554 | ENSG00000239732 |
| ENSG00000104689 | ENSG00000128422 | ENSG00000141564 | ENSG00000167965 | ENSG00000187796 | ENSG00000271503 |
| ENSG00000105141 | ENSG00000134460 | ENSG00000142208 | ENSG00000168685 | ENSG00000196664 | ENSG00000274233 |
