# Supplementary material for: Identification of sequences common to more than one therapeutic target to treat complex diseases: simulating the high variance in sequence interactivity evolved to modulate robust phenotypes
Source: BMC Genomics. 2015 Jul 18;16(1):530. doi: 10.1186/s12864-015-1727-6 (PMC4506634; doi:10.1186/s12864-015-1727-6)
Supplement: Additional file 5: Table S5. — Accession numbers of peptides that were searched for targets shared by key pathways cancer and immune diseases to facilitate the design of new multispecific methods to treat complex diseases. [file 12864_2015_1727_MOESM5_ESM.docx]

**Table S5 Accession numbers of peptides that were searched for targets shared by key pathways cancer and immune diseases to facilitate the design of new multispecific methods and treat complex diseases.**

| Analyzed peptide sequences related to cancer | | |  |  |  |
| --- | --- | --- | --- | --- | --- |
| ENSP00000009180 | ENSP00000328213 | ENSP00000367038 | ENSP00000401599 | ENSP00000428695 | ENSP00000451634 |
| ENSP00000011898 | ENSP00000329425 | ENSP00000367044 | ENSP00000401888 | ENSP00000428703 | ENSP00000451824 |
| ENSP00000166244 | ENSP00000329623 | ENSP00000368458 | ENSP00000401916 | ENSP00000428884 | ENSP00000451828 |
| ENSP00000168712 | ENSP00000330161 | ENSP00000369222 | ENSP00000402103 | ENSP00000428924 | ENSP00000451856 |
| ENSP00000180166 | ENSP00000331504 | ENSP00000369223 | ENSP00000402847 | ENSP00000428983 | ENSP00000451919 |
| ENSP00000188790 | ENSP00000332118 | ENSP00000370374 | ENSP00000403204 | ENSP00000428988 | ENSP00000451958 |
| ENSP00000189444 | ENSP00000332454 | ENSP00000370912 | ENSP00000403392 | ENSP00000429021 | ENSP00000452120 |
| ENSP00000189978 | ENSP00000332659 | ENSP00000371067 | ENSP00000403459 | ENSP00000429044 | ENSP00000452240 |
| ENSP00000212015 | ENSP00000334122 | ENSP00000371341 | ENSP00000403730 | ENSP00000429055 | ENSP00000452512 |
| ENSP00000215530 | ENSP00000334346 | ENSP00000372881 | ENSP00000403842 | ENSP00000429089 | ENSP00000452566 |
| ENSP00000215832 | ENSP00000335062 | ENSP00000373083 | ENSP00000403890 | ENSP00000429218 | ENSP00000452574 |
| ENSP00000217169 | ENSP00000335096 | ENSP00000373570 | ENSP00000404047 | ENSP00000429259 | ENSP00000452603 |
| ENSP00000217188 | ENSP00000335250 | ENSP00000373700 | ENSP00000404083 | ENSP00000429289 | ENSP00000452995 |
| ENSP00000220003 | ENSP00000336752 | ENSP00000373854 | ENSP00000404089 | ENSP00000429294 | ENSP00000453835 |
| ENSP00000221132 | ENSP00000337247 | ENSP00000373923 | ENSP00000404219 | ENSP00000429590 | ENSP00000454050 |
| ENSP00000221930 | ENSP00000337451 | ENSP00000374323 | ENSP00000404524 | ENSP00000429642 | ENSP00000454089 |
| ENSP00000222157 | ENSP00000337612 | ENSP00000374663 | ENSP00000404786 | ENSP00000429692 | ENSP00000454130 |
| ENSP00000222390 | ENSP00000337665 | ENSP00000375719 | ENSP00000405158 | ENSP00000429751 | ENSP00000454146 |
| ENSP00000222747 | ENSP00000337675 | ENSP00000375811 | ENSP00000405529 | ENSP00000429813 | ENSP00000454342 |
| ENSP00000223095 | ENSP00000337825 | ENSP00000375891 | ENSP00000405564 | ENSP00000429848 | ENSP00000454568 |
| ENSP00000226574 | ENSP00000338200 | ENSP00000375892 | ENSP00000405798 | ENSP00000429866 | ENSP00000454623 |
| ENSP00000227507 | ENSP00000338235 | ENSP00000376120 | ENSP00000405865 | ENSP00000429985 | ENSP00000454640 |
| ENSP00000227758 | ENSP00000338983 | ENSP00000376637 | ENSP00000405926 | ENSP00000429992 | ENSP00000454906 |
| ENSP00000229030 | ENSP00000339393 | ENSP00000376638 | ENSP00000406249 | ENSP00000430026 | ENSP00000454908 |
| ENSP00000229471 | ENSP00000339686 | ENSP00000376971 | ENSP00000406359 | ENSP00000430060 | ENSP00000455441 |
| ENSP00000230381 | ENSP00000339690 | ENSP00000377003 | ENSP00000406533 | ENSP00000430257 | ENSP00000456008 |
| ENSP00000231803 | ENSP00000339824 | ENSP00000377016 | ENSP00000407586 | ENSP00000430327 | ENSP00000456521 |
| ENSP00000232375 | ENSP00000340039 | ENSP00000377028 | ENSP00000407670 | ENSP00000430364 | ENSP00000456888 |
| ENSP00000233057 | ENSP00000340149 | ENSP00000377029 | ENSP00000407926 | ENSP00000430455 | ENSP00000457497 |
| ENSP00000233573 | ENSP00000340361 | ENSP00000377062 | ENSP00000407999 | ENSP00000430486 | ENSP00000457714 |
| ENSP00000234091 | ENSP00000340636 | ENSP00000377064 | ENSP00000408145 | ENSP00000430608 | ENSP00000457785 |
| ENSP00000237837 | ENSP00000340690 | ENSP00000377065 | ENSP00000408198 | ENSP00000430715 | ENSP00000458111 |
| ENSP00000239165 | ENSP00000340742 | ENSP00000377067 | ENSP00000408340 | ENSP00000430824 | ENSP00000458739 |
| ENSP00000240093 | ENSP00000341325 | ENSP00000377068 | ENSP00000408347 | ENSP00000430875 | ENSP00000461006 |
| ENSP00000241393 | ENSP00000342235 | ENSP00000377140 | ENSP00000408695 | ENSP00000430906 | ENSP00000461850 |
| ENSP00000241453 | ENSP00000342307 | ENSP00000377254 | ENSP00000408720 | ENSP00000430966 | ENSP00000462022 |
| ENSP00000246043 | ENSP00000342376 | ENSP00000377259 | ENSP00000408835 | ENSP00000430988 | ENSP00000462024 |
| ENSP00000247207 | ENSP00000342814 | ENSP00000377291 | ENSP00000408907 | ENSP00000431041 | ENSP00000462270 |
| ENSP00000247829 | ENSP00000343204 | ENSP00000377754 | ENSP00000409061 | ENSP00000431054 | ENSP00000462315 |
| ENSP00000248553 | ENSP00000343741 | ENSP00000377759 | ENSP00000409142 | ENSP00000431085 | ENSP00000462438 |
| ENSP00000249749 | ENSP00000343952 | ENSP00000377761 | ENSP00000409151 | ENSP00000431110 | ENSP00000462468 |
| ENSP00000250405 | ENSP00000344306 | ENSP00000377836 | ENSP00000409208 | ENSP00000431389 | ENSP00000462469 |
| ENSP00000251157 | ENSP00000344456 | ENSP00000377837 | ENSP00000409227 | ENSP00000431418 | ENSP00000462715 |
| ENSP00000251849 | ENSP00000344798 | ENSP00000377839 | ENSP00000409302 | ENSP00000431445 | ENSP00000462776 |
| ENSP00000253055 | ENSP00000344936 | ENSP00000377880 | ENSP00000409377 | ENSP00000431506 | ENSP00000462808 |
| ENSP00000255448 | ENSP00000345203 | ENSP00000377989 | ENSP00000409462 | ENSP00000431517 | ENSP00000462919 |
| ENSP00000256078 | ENSP00000345213 | ENSP00000378297 | ENSP00000409707 | ENSP00000431723 | ENSP00000463002 |
| ENSP00000257290 | ENSP00000345524 | ENSP00000378481 | ENSP00000409761 | ENSP00000431885 | ENSP00000463086 |
| ENSP00000257904 | ENSP00000345571 | ENSP00000378485 | ENSP00000410031 | ENSP00000432068 | ENSP00000463126 |
| ENSP00000258148 | ENSP00000345785 | ENSP00000378559 | ENSP00000410070 | ENSP00000432082 | ENSP00000463262 |
| ENSP00000258149 | ENSP00000345973 | ENSP00000378625 | ENSP00000410158 | ENSP00000432083 | ENSP00000463368 |
| ENSP00000259089 | ENSP00000346643 | ENSP00000378626 | ENSP00000410256 | ENSP00000432292 | ENSP00000463416 |
| ENSP00000260442 | ENSP00000346671 | ENSP00000378628 | ENSP00000410294 | ENSP00000432320 | ENSP00000463427 |
| ENSP00000260731 | ENSP00000346899 | ENSP00000378717 | ENSP00000410477 | ENSP00000432410 | ENSP00000463686 |
| ENSP00000260795 | ENSP00000347293 | ENSP00000379131 | ENSP00000410559 | ENSP00000432479 | ENSP00000463714 |
| ENSP00000260943 | ENSP00000347858 | ENSP00000379353 | ENSP00000410675 | ENSP00000432693 | ENSP00000463719 |
| ENSP00000261254 | ENSP00000347942 | ENSP00000379358 | ENSP00000410694 | ENSP00000432728 | ENSP00000463806 |
| ENSP00000261479 | ENSP00000348089 | ENSP00000379359 | ENSP00000410953 | ENSP00000432742 | ENSP00000463891 |
| ENSP00000261601 | ENSP00000348537 | ENSP00000379437 | ENSP00000410980 | ENSP00000432890 | ENSP00000464252 |
| ENSP00000261793 | ENSP00000348559 | ENSP00000379486 | ENSP00000411226 | ENSP00000432972 | ENSP00000464380 |
| ENSP00000261799 | ENSP00000348637 | ENSP00000379488 | ENSP00000411809 | ENSP00000433163 | ENSP00000464420 |
| ENSP00000261937 | ENSP00000348916 | ENSP00000379585 | ENSP00000412219 | ENSP00000433203 | ENSP00000464447 |
| ENSP00000262052 | ENSP00000349252 | ENSP00000380227 | ENSP00000412265 | ENSP00000433569 | ENSP00000465291 |
| ENSP00000262067 | ENSP00000349762 | ENSP00000380262 | ENSP00000412504 | ENSP00000433639 | ENSP00000465534 |
| ENSP00000262407 | ENSP00000350166 | ENSP00000380280 | ENSP00000412903 | ENSP00000433663 | ENSP00000465556 |
| ENSP00000262651 | ENSP00000350886 | ENSP00000380292 | ENSP00000412957 | ENSP00000433746 | ENSP00000465661 |
| ENSP00000262715 | ENSP00000350941 | ENSP00000380297 | ENSP00000413090 | ENSP00000433816 | ENSP00000465761 |
| ENSP00000263025 | ENSP00000351059 | ENSP00000380302 | ENSP00000413151 | ENSP00000433851 | ENSP00000465868 |
| ENSP00000263087 | ENSP00000351095 | ENSP00000380860 | ENSP00000413354 | ENSP00000433886 | ENSP00000466041 |
| ENSP00000263451 | ENSP00000351123 | ENSP00000381097 | ENSP00000413496 | ENSP00000434034 | ENSP00000466063 |
| ENSP00000263645 | ENSP00000351172 | ENSP00000381185 | ENSP00000413583 | ENSP00000434473 | ENSP00000466233 |
| ENSP00000263734 | ENSP00000351276 | ENSP00000381412 | ENSP00000413843 | ENSP00000434525 | ENSP00000466340 |
| ENSP00000263735 | ENSP00000351383 | ENSP00000381577 | ENSP00000413857 | ENSP00000434557 | ENSP00000466417 |
| ENSP00000263798 | ENSP00000351486 | ENSP00000381762 | ENSP00000414629 | ENSP00000434708 | ENSP00000466617 |
| ENSP00000263800 | ENSP00000351995 | ENSP00000381775 | ENSP00000414764 | ENSP00000434712 | ENSP00000466675 |
| ENSP00000263817 | ENSP00000352135 | ENSP00000381803 | ENSP00000414775 | ENSP00000434800 | ENSP00000466927 |
| ENSP00000263923 | ENSP00000352309 | ENSP00000382126 | ENSP00000414792 | ENSP00000434831 | ENSP00000467000 |
| ENSP00000263932 | ENSP00000352398 | ENSP00000382166 | ENSP00000414914 | ENSP00000434869 | ENSP00000467006 |
| ENSP00000264107 | ENSP00000352414 | ENSP00000382342 | ENSP00000415126 | ENSP00000434878 | ENSP00000467173 |
| ENSP00000264234 | ENSP00000352516 | ENSP00000382915 | ENSP00000415139 | ENSP00000434931 | ENSP00000467259 |
| ENSP00000264246 | ENSP00000352901 | ENSP00000383053 | ENSP00000415516 | ENSP00000434979 | ENSP00000467269 |
| ENSP00000264316 | ENSP00000353262 | ENSP00000383125 | ENSP00000415559 | ENSP00000435237 | ENSP00000467288 |
| ENSP00000264657 | ENSP00000353433 | ENSP00000383234 | ENSP00000415753 | ENSP00000435254 | ENSP00000467336 |
| ENSP00000264664 | ENSP00000353611 | ENSP00000383715 | ENSP00000415949 | ENSP00000435283 | ENSP00000467472 |
| ENSP00000264741 | ENSP00000353824 | ENSP00000384063 | ENSP00000416058 | ENSP00000435371 | ENSP00000467977 |
| ENSP00000264818 | ENSP00000353846 | ENSP00000384293 | ENSP00000416811 | ENSP00000435605 | ENSP00000467985 |
| ENSP00000264972 | ENSP00000354045 | ENSP00000384446 | ENSP00000417030 | ENSP00000435687 | ENSP00000467992 |
| ENSP00000265131 | ENSP00000354492 | ENSP00000384508 | ENSP00000417216 | ENSP00000435689 | ENSP00000468030 |
| ENSP00000265723 | ENSP00000354558 | ENSP00000384943 | ENSP00000417281 | ENSP00000435811 | ENSP00000468465 |
| ENSP00000265724 | ENSP00000354607 | ENSP00000385014 | ENSP00000417435 | ENSP00000435970 | ENSP00000468576 |
| ENSP00000266434 | ENSP00000355755 | ENSP00000385185 | ENSP00000417779 | ENSP00000436155 | ENSP00000469340 |
| ENSP00000266624 | ENSP00000355757 | ENSP00000385326 | ENSP00000418033 | ENSP00000436175 | ENSP00000470604 |
| ENSP00000267101 | ENSP00000355759 | ENSP00000385465 | ENSP00000418352 | ENSP00000436288 | ENSP00000470822 |
| ENSP00000268035 | ENSP00000355896 | ENSP00000385476 | ENSP00000419060 | ENSP00000436425 | ENSP00000470826 |
| ENSP00000268296 | ENSP00000355897 | ENSP00000385565 | ENSP00000419080 | ENSP00000436554 | ENSP00000471116 |
| ENSP00000269571 | ENSP00000355965 | ENSP00000385604 | ENSP00000419190 | ENSP00000436741 | ENSP00000471369 |
| ENSP00000270202 | ENSP00000356147 | ENSP00000386276 | ENSP00000419470 | ENSP00000436772 | ENSP00000471497 |
| ENSP00000271324 | ENSP00000356148 | ENSP00000386761 | ENSP00000419574 | ENSP00000436804 | ENSP00000471586 |
| ENSP00000272602 | ENSP00000356150 | ENSP00000386829 | ENSP00000419688 | ENSP00000436826 | ENSP00000471916 |
| ENSP00000273783 | ENSP00000356151 | ENSP00000386884 | ENSP00000419711 | ENSP00000437214 | ENSP00000472371 |
| ENSP00000273854 | ENSP00000356418 | ENSP00000387140 | ENSP00000420119 | ENSP00000437631 | ENSP00000472382 |
| ENSP00000274254 | ENSP00000356419 | ENSP00000387455 | ENSP00000420338 | ENSP00000437804 | ENSP00000472767 |
| ENSP00000274255 | ENSP00000356420 | ENSP00000388129 | ENSP00000420457 | ENSP00000437841 | ENSP00000473021 |
| ENSP00000274625 | ENSP00000356421 | ENSP00000388648 | ENSP00000420533 | ENSP00000437955 | ENSP00000473299 |
| ENSP00000275493 | ENSP00000356424 | ENSP00000389028 | ENSP00000420598 | ENSP00000438460 | ENSP00000473563 |
| ENSP00000276052 | ENSP00000356425 | ENSP00000389208 | ENSP00000420904 | ENSP00000438482 | ENSP00000474011 |
| ENSP00000276431 | ENSP00000356438 | ENSP00000389338 | ENSP00000421155 | ENSP00000439140 | ENSP00000474109 |
| ENSP00000278833 | ENSP00000356898 | ENSP00000389563 | ENSP00000421174 | ENSP00000439231 | ENSP00000476145 |
| ENSP00000280892 | ENSP00000356899 | ENSP00000389624 | ENSP00000421241 | ENSP00000439238 | ENSP00000476483 |
| ENSP00000281092 | ENSP00000357179 | ENSP00000390088 | ENSP00000421260 | ENSP00000439894 | ENSP00000477613 |
| ENSP00000281821 | ENSP00000357374 | ENSP00000390435 | ENSP00000421287 | ENSP00000440063 | ENSP00000477624 |
| ENSP00000282397 | ENSP00000357375 | ENSP00000390650 | ENSP00000421353 | ENSP00000440237 | ENSP00000477645 |
| ENSP00000282588 | ENSP00000357377 | ENSP00000390661 | ENSP00000421535 | ENSP00000440274 | ENSP00000477713 |
| ENSP00000286201 | ENSP00000357378 | ENSP00000390987 | ENSP00000421691 | ENSP00000440646 | ENSP00000477734 |
| ENSP00000286301 | ENSP00000357381 | ENSP00000391153 | ENSP00000421711 | ENSP00000440842 | ENSP00000477807 |
| ENSP00000287497 | ENSP00000357383 | ENSP00000391265 | ENSP00000421870 | ENSP00000441035 | ENSP00000478031 |
| ENSP00000287934 | ENSP00000357642 | ENSP00000391310 | ENSP00000421938 | ENSP00000441169 | ENSP00000478080 |
| ENSP00000288135 | ENSP00000357643 | ENSP00000391517 | ENSP00000421951 | ENSP00000441691 | ENSP00000478148 |
| ENSP00000288602 | ENSP00000357656 | ENSP00000392196 | ENSP00000422095 | ENSP00000441822 | ENSP00000478255 |
| ENSP00000289013 | ENSP00000357667 | ENSP00000392253 | ENSP00000422139 | ENSP00000441841 | ENSP00000478324 |
| ENSP00000290277 | ENSP00000357671 | ENSP00000392416 | ENSP00000422145 | ENSP00000442070 | ENSP00000478537 |
| ENSP00000290573 | ENSP00000358022 | ENSP00000392458 | ENSP00000422169 | ENSP00000442309 | ENSP00000478581 |
| ENSP00000291906 | ENSP00000358052 | ENSP00000392546 | ENSP00000422212 | ENSP00000442317 | ENSP00000478620 |
| ENSP00000292174 | ENSP00000358054 | ENSP00000392645 | ENSP00000422553 | ENSP00000442630 | ENSP00000478847 |
| ENSP00000292408 | ENSP00000358055 | ENSP00000392662 | ENSP00000422798 | ENSP00000442807 | ENSP00000479013 |
| ENSP00000292432 | ENSP00000358056 | ENSP00000392696 | ENSP00000422889 | ENSP00000443149 | ENSP00000479024 |
| ENSP00000293379 | ENSP00000358057 | ENSP00000392983 | ENSP00000423325 | ENSP00000443311 | ENSP00000479215 |
| ENSP00000294312 | ENSP00000358211 | ENSP00000393294 | ENSP00000423481 | ENSP00000443450 | ENSP00000479472 |
| ENSP00000295025 | ENSP00000358303 | ENSP00000393312 | ENSP00000423877 | ENSP00000443816 | ENSP00000479618 |
| ENSP00000295854 | ENSP00000358309 | ENSP00000393590 | ENSP00000423950 | ENSP00000443897 | ENSP00000479620 |
| ENSP00000296474 | ENSP00000358310 | ENSP00000393596 | ENSP00000423977 | ENSP00000444028 | ENSP00000479709 |
| ENSP00000296550 | ENSP00000358525 | ENSP00000393608 | ENSP00000424070 | ENSP00000444085 | ENSP00000479777 |
| ENSP00000296585 | ENSP00000358529 | ENSP00000393707 | ENSP00000424218 | ENSP00000444095 | ENSP00000480088 |
| ENSP00000297185 | ENSP00000358548 | ENSP00000393803 | ENSP00000424355 | ENSP00000444174 | ENSP00000480206 |
| ENSP00000298649 | ENSP00000358656 | ENSP00000393921 | ENSP00000424365 | ENSP00000444430 | ENSP00000480211 |
| ENSP00000299252 | ENSP00000358983 | ENSP00000394297 | ENSP00000424397 | ENSP00000444541 | ENSP00000480684 |
| ENSP00000299752 | ENSP00000358990 | ENSP00000394511 | ENSP00000424567 | ENSP00000444566 | ENSP00000480686 |
| ENSP00000299767 | ENSP00000358991 | ENSP00000394560 | ENSP00000424632 | ENSP00000444597 | ENSP00000480763 |
| ENSP00000300093 | ENSP00000358997 | ENSP00000394960 | ENSP00000424638 | ENSP00000444799 | ENSP00000480778 |
| ENSP00000300305 | ENSP00000359478 | ENSP00000394999 | ENSP00000424642 | ENSP00000444844 | ENSP00000480836 |
| ENSP00000300738 | ENSP00000360242 | ENSP00000395154 | ENSP00000424670 | ENSP00000444928 | ENSP00000481084 |
| ENSP00000301178 | ENSP00000360901 | ENSP00000395160 | ENSP00000424790 | ENSP00000444986 | ENSP00000481134 |
| ENSP00000301633 | ENSP00000361072 | ENSP00000395164 | ENSP00000424815 | ENSP00000445030 | ENSP00000481231 |
| ENSP00000302665 | ENSP00000361359 | ENSP00000395230 | ENSP00000424905 | ENSP00000445222 | ENSP00000481464 |
| ENSP00000302955 | ENSP00000361423 | ENSP00000395243 | ENSP00000424960 | ENSP00000445282 | ENSP00000481526 |
| ENSP00000302961 | ENSP00000361554 | ENSP00000395254 | ENSP00000425003 | ENSP00000446466 | ENSP00000481616 |
| ENSP00000302993 | ENSP00000362111 | ENSP00000395425 | ENSP00000425132 | ENSP00000446763 | ENSP00000481994 |
| ENSP00000303887 | ENSP00000362139 | ENSP00000395588 | ENSP00000425199 | ENSP00000447005 | ENSP00000481996 |
| ENSP00000303939 | ENSP00000362387 | ENSP00000395716 | ENSP00000425232 | ENSP00000447160 | ENSP00000482073 |
| ENSP00000307701 | ENSP00000362608 | ENSP00000395917 | ENSP00000425456 | ENSP00000447274 | ENSP00000482388 |
| ENSP00000308495 | ENSP00000362658 | ENSP00000396270 | ENSP00000425561 | ENSP00000447347 | ENSP00000482407 |
| ENSP00000308734 | ENSP00000362659 | ENSP00000396486 | ENSP00000425626 | ENSP00000447510 | ENSP00000482479 |
| ENSP00000309181 | ENSP00000362663 | ENSP00000396532 | ENSP00000425648 | ENSP00000447730 | ENSP00000482642 |
| ENSP00000309428 | ENSP00000362665 | ENSP00000396840 | ENSP00000425897 | ENSP00000447779 | ENSP00000482735 |
| ENSP00000309572 | ENSP00000362668 | ENSP00000396864 | ENSP00000425902 | ENSP00000448098 | ENSP00000482827 |
| ENSP00000309597 | ENSP00000362680 | ENSP00000396968 | ENSP00000426042 | ENSP00000448483 | ENSP00000482886 |
| ENSP00000309878 | ENSP00000362795 | ENSP00000397258 | ENSP00000426057 | ENSP00000448636 | ENSP00000483062 |
| ENSP00000309973 | ENSP00000362797 | ENSP00000397422 | ENSP00000426147 | ENSP00000448671 | ENSP00000483176 |
| ENSP00000310036 | ENSP00000363115 | ENSP00000397699 | ENSP00000426472 | ENSP00000448729 | ENSP00000483213 |
| ENSP00000310219 | ENSP00000363116 | ENSP00000397746 | ENSP00000426489 | ENSP00000448885 | ENSP00000483316 |
| ENSP00000310742 | ENSP00000363117 | ENSP00000398140 | ENSP00000426492 | ENSP00000448946 | ENSP00000483860 |
| ENSP00000310771 | ENSP00000363561 | ENSP00000398316 | ENSP00000426595 | ENSP00000448963 | ENSP00000484033 |
| ENSP00000314620 | ENSP00000363562 | ENSP00000398318 | ENSP00000426602 | ENSP00000449129 | ENSP00000484039 |
| ENSP00000315130 | ENSP00000363563 | ENSP00000398337 | ENSP00000426607 | ENSP00000449138 | ENSP00000484154 |
| ENSP00000315190 | ENSP00000363571 | ENSP00000398489 | ENSP00000426917 | ENSP00000449179 | ENSP00000484385 |
| ENSP00000315644 | ENSP00000363758 | ENSP00000398591 | ENSP00000427000 | ENSP00000449391 | ENSP00000484420 |
| ENSP00000316395 | ENSP00000363761 | ENSP00000398614 | ENSP00000427222 | ENSP00000449713 | ENSP00000484452 |
| ENSP00000316889 | ENSP00000363763 | ENSP00000398655 | ENSP00000427235 | ENSP00000449725 | ENSP00000484553 |
| ENSP00000317272 | ENSP00000363775 | ENSP00000398776 | ENSP00000427289 | ENSP00000450267 | ENSP00000484724 |
| ENSP00000317859 | ENSP00000363822 | ENSP00000398960 | ENSP00000427545 | ENSP00000450475 | ENSP00000484767 |
| ENSP00000319486 | ENSP00000363826 | ENSP00000399324 | ENSP00000427580 | ENSP00000450508 | ENSP00000484832 |
| ENSP00000320147 | ENSP00000364086 | ENSP00000399419 | ENSP00000427638 | ENSP00000450528 | ENSP00000484838 |
| ENSP00000321410 | ENSP00000364802 | ENSP00000399532 | ENSP00000427757 | ENSP00000450541 | ENSP00000484892 |
| ENSP00000321797 | ENSP00000364805 | ENSP00000399926 | ENSP00000427828 | ENSP00000450681 | ENSP00000485776 |
| ENSP00000321945 | ENSP00000364898 | ENSP00000399974 | ENSP00000427993 | ENSP00000450688 | ENSP00000485817 |
| ENSP00000323315 | ENSP00000364899 | ENSP00000400162 | ENSP00000427999 | ENSP00000450830 | ENSP00000485852 |
| ENSP00000323901 | ENSP00000364904 | ENSP00000400309 | ENSP00000428042 | ENSP00000450844 | ENSP00000486048 |
| ENSP00000324173 | ENSP00000364907 | ENSP00000400475 | ENSP00000428045 | ENSP00000450928 | ENSP00000486536 |
| ENSP00000324180 | ENSP00000365012 | ENSP00000400508 | ENSP00000428168 | ENSP00000451166 | ENSP00000486627 |
| ENSP00000324443 | ENSP00000365022 | ENSP00000400637 | ENSP00000428313 | ENSP00000451171 | ENSP00000486858 |
| ENSP00000324740 | ENSP00000365223 | ENSP00000400708 | ENSP00000428349 | ENSP00000451254 |  |
| ENSP00000325875 | ENSP00000365280 | ENSP00000400868 | ENSP00000428351 | ENSP00000451290 |  |
| ENSP00000327229 | ENSP00000365313 | ENSP00000401088 | ENSP00000428424 | ENSP00000451409 |  |
| ENSP00000327290 | ENSP00000366238 | ENSP00000401206 | ENSP00000428493 | ENSP00000451432 |  |
| ENSP00000327293 | ENSP00000366244 | ENSP00000401405 | ENSP00000428508 | ENSP00000451470 |  |
| Analyzed peptide sequences related to immunological diseases | | | |  |  |
| ENSP00000162749 | ENSP00000340589 | ENSP00000380878 | ENSP00000414867 | ENSP00000436875 | ENSP00000458303 |
| ENSP00000170630 | ENSP00000343147 | ENSP00000382800 | ENSP00000415148 | ENSP00000437709 | ENSP00000459612 |
| ENSP00000189444 | ENSP00000343477 | ENSP00000384293 | ENSP00000416815 | ENSP00000438152 | ENSP00000459661 |
| ENSP00000216223 | ENSP00000344132 | ENSP00000384928 | ENSP00000420975 | ENSP00000438343 | ENSP00000459701 |
| ENSP00000218032 | ENSP00000345601 | ENSP00000385043 | ENSP00000421207 | ENSP00000439981 | ENSP00000460136 |
| ENSP00000221132 | ENSP00000346390 | ENSP00000385227 | ENSP00000421259 | ENSP00000440643 | ENSP00000460216 |
| ENSP00000223423 | ENSP00000347426 | ENSP00000385326 | ENSP00000421483 | ENSP00000441667 | ENSP00000460475 |
| ENSP00000226730 | ENSP00000347979 | ENSP00000385458 | ENSP00000421632 | ENSP00000441691 | ENSP00000460940 |
| ENSP00000229135 | ENSP00000349896 | ENSP00000385675 | ENSP00000421856 | ENSP00000441751 | ENSP00000461268 |
| ENSP00000231228 | ENSP00000349932 | ENSP00000385718 | ENSP00000422453 | ENSP00000441803 | ENSP00000461607 |
| ENSP00000240139 | ENSP00000350016 | ENSP00000387173 | ENSP00000422781 | ENSP00000442029 | ENSP00000461850 |
| ENSP00000256452 | ENSP00000353874 | ENSP00000387210 | ENSP00000422990 | ENSP00000442059 | ENSP00000465185 |
| ENSP00000256876 | ENSP00000353881 | ENSP00000387354 | ENSP00000423017 | ENSP00000442242 | ENSP00000465443 |
| ENSP00000258743 | ENSP00000354459 | ENSP00000387640 | ENSP00000423036 | ENSP00000442405 | ENSP00000466166 |
| ENSP00000259206 | ENSP00000354558 | ENSP00000388858 | ENSP00000423075 | ENSP00000442479 | ENSP00000467398 |
| ENSP00000260010 | ENSP00000354612 | ENSP00000389265 | ENSP00000423184 | ENSP00000442919 | ENSP00000467418 |
| ENSP00000262467 | ENSP00000354816 | ENSP00000389490 | ENSP00000423326 | ENSP00000443469 | ENSP00000467537 |
| ENSP00000262963 | ENSP00000354932 | ENSP00000389492 | ENSP00000423668 | ENSP00000443478 | ENSP00000468455 |
| ENSP00000263341 | ENSP00000355846 | ENSP00000389600 | ENSP00000423725 | ENSP00000443897 | ENSP00000468672 |
| ENSP00000264867 | ENSP00000356438 | ENSP00000390753 | ENSP00000423755 | ENSP00000444095 | ENSP00000468803 |
| ENSP00000265131 | ENSP00000356579 | ENSP00000391274 | ENSP00000424080 | ENSP00000445380 | ENSP00000469439 |
| ENSP00000265164 | ENSP00000357470 | ENSP00000392059 | ENSP00000424113 | ENSP00000449109 | ENSP00000470826 |
| ENSP00000265724 | ENSP00000358043 | ENSP00000392348 | ENSP00000424181 | ENSP00000449169 | ENSP00000471586 |
| ENSP00000268296 | ENSP00000358045 | ENSP00000392698 | ENSP00000424718 | ENSP00000450681 | ENSP00000471966 |
| ENSP00000269280 | ENSP00000358983 | ENSP00000392858 | ENSP00000424894 | ENSP00000450688 | ENSP00000473675 |
| ENSP00000269907 | ENSP00000359338 | ENSP00000393417 | ENSP00000424923 | ENSP00000450751 | ENSP00000474141 |
| ENSP00000270202 | ENSP00000359345 | ENSP00000398022 | ENSP00000425159 | ENSP00000450985 | ENSP00000474412 |
| ENSP00000271630 | ENSP00000360797 | ENSP00000398117 | ENSP00000425309 | ENSP00000451166 | ENSP00000475057 |
| ENSP00000276431 | ENSP00000360799 | ENSP00000398698 | ENSP00000425538 | ENSP00000451290 | ENSP00000477739 |
| ENSP00000285333 | ENSP00000360843 | ENSP00000398745 | ENSP00000426069 | ENSP00000451368 | ENSP00000477921 |
| ENSP00000287497 | ENSP00000362802 | ENSP00000399099 | ENSP00000426426 | ENSP00000451470 | ENSP00000477997 |
| ENSP00000289963 | ENSP00000363089 | ENSP00000399419 | ENSP00000426565 | ENSP00000451824 | ENSP00000478206 |
| ENSP00000295025 | ENSP00000365290 | ENSP00000400400 | ENSP00000427606 | ENSP00000451828 | ENSP00000478255 |
| ENSP00000295683 | ENSP00000366034 | ENSP00000400416 | ENSP00000427669 | ENSP00000454130 | ENSP00000478339 |
| ENSP00000296435 | ENSP00000368436 | ENSP00000400854 | ENSP00000427688 | ENSP00000454235 | ENSP00000478516 |
| ENSP00000301724 | ENSP00000369287 | ENSP00000401020 | ENSP00000427827 | ENSP00000454568 | ENSP00000478985 |
| ENSP00000304915 | ENSP00000369293 | ENSP00000401608 | ENSP00000427999 | ENSP00000454623 | ENSP00000479729 |
| ENSP00000306157 | ENSP00000370034 | ENSP00000402024 | ENSP00000428358 | ENSP00000454629 | ENSP00000479835 |
| ENSP00000307272 | ENSP00000370878 | ENSP00000402685 | ENSP00000428372 | ENSP00000454684 | ENSP00000479894 |
| ENSP00000308051 | ENSP00000371376 | ENSP00000403260 | ENSP00000428390 | ENSP00000454698 | ENSP00000480211 |
| ENSP00000308452 | ENSP00000371888 | ENSP00000403731 | ENSP00000428884 | ENSP00000454725 | ENSP00000480266 |
| ENSP00000308925 | ENSP00000372988 | ENSP00000405150 | ENSP00000428929 | ENSP00000455046 | ENSP00000480778 |
| ENSP00000309196 | ENSP00000373358 | ENSP00000407044 | ENSP00000429379 | ENSP00000455458 | ENSP00000481498 |
| ENSP00000311032 | ENSP00000373854 | ENSP00000407142 | ENSP00000430555 | ENSP00000455475 | ENSP00000481526 |
| ENSP00000312082 | ENSP00000375685 | ENSP00000407219 | ENSP00000430783 | ENSP00000455632 | ENSP00000483359 |
| ENSP00000315768 | ENSP00000377210 | ENSP00000409395 | ENSP00000431619 | ENSP00000455642 | ENSP00000483540 |
| ENSP00000317859 | ENSP00000377213 | ENSP00000409680 | ENSP00000431947 | ENSP00000455714 | ENSP00000483860 |
| ENSP00000319635 | ENSP00000377989 | ENSP00000410076 | ENSP00000432340 | ENSP00000455850 | ENSP00000484575 |
| ENSP00000320580 | ENSP00000377997 | ENSP00000410256 | ENSP00000432910 | ENSP00000456046 | ENSP00000484692 |
| ENSP00000321345 | ENSP00000378305 | ENSP00000410506 | ENSP00000433138 | ENSP00000456063 | ENSP00000484800 |
| ENSP00000324366 | ENSP00000378306 | ENSP00000410668 | ENSP00000434250 | ENSP00000456076 | ENSP00000484921 |
| ENSP00000326759 | ENSP00000378322 | ENSP00000411406 | ENSP00000434259 | ENSP00000456248 | ENSP00000485876 |
| ENSP00000327313 | ENSP00000378323 | ENSP00000411606 | ENSP00000434303 | ENSP00000456405 | ENSP00000486645 |
| ENSP00000327890 | ENSP00000378652 | ENSP00000412209 | ENSP00000434599 | ENSP00000456669 | ENSP00000486667 |
| ENSP00000329072 | ENSP00000379111 | ENSP00000412460 | ENSP00000434779 | ENSP00000456930 |  |
| ENSP00000337733 | ENSP00000379457 | ENSP00000413143 | ENSP00000434929 | ENSP00000456944 |  |
| ENSP00000339553 | ENSP00000380313 | ENSP00000413224 | ENSP00000435536 | ENSP00000457131 |  |
| ENSP00000339804 | ENSP00000380389 | ENSP00000413686 | ENSP00000435808 | ENSP00000457870 |  |
