# Supplementary material for: Identification of sequences common to more than one therapeutic target to treat complex diseases: simulating the high variance in sequence interactivity evolved to modulate robust phenotypes
Source: BMC Genomics. 2015 Jul 18;16(1):530. doi: 10.1186/s12864-015-1727-6 (PMC4506634; doi:10.1186/s12864-015-1727-6)
Supplement: Additional file 6: Figure S1. — Identification of sequences shared by multiple genes to form a library of nucleotide- and peptide-based tools capable of simultaneously targeting key pathways. [file 12864_2015_1727_MOESM6_ESM.docx]

**Figure S1 Identification of sequences shared by multiple genes to form a library of nucleotide- and peptide-based tools capable of simultaneously targeting key cancer pathways or other diseases with a strong genetic background.** Rather than searching for conserved protein domains and functional sites using alignment algorithms, the search was performed by dividing each cDNA or protein of interest into a matrix of multiple cells of 12 nucleotides or 4 amino acids (A), respectively, and then, a script was run to search for repeated cells (B). This method increases the sensitivity of the search by removing the negative influence of non-common flanking sequences in alignment algorithms. Sequences were searched from a collection of 308 gene and 1105 peptide sequences related to cancer, and 72 gene and 344 peptide sequences related to immune disorders.

**A**


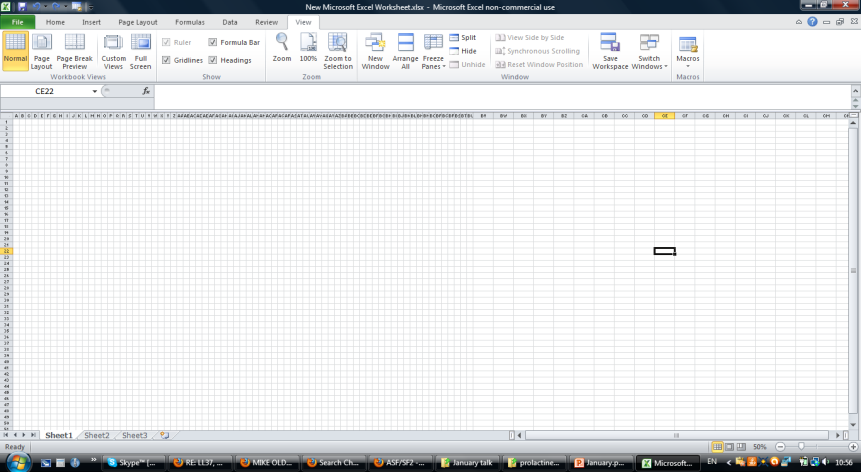

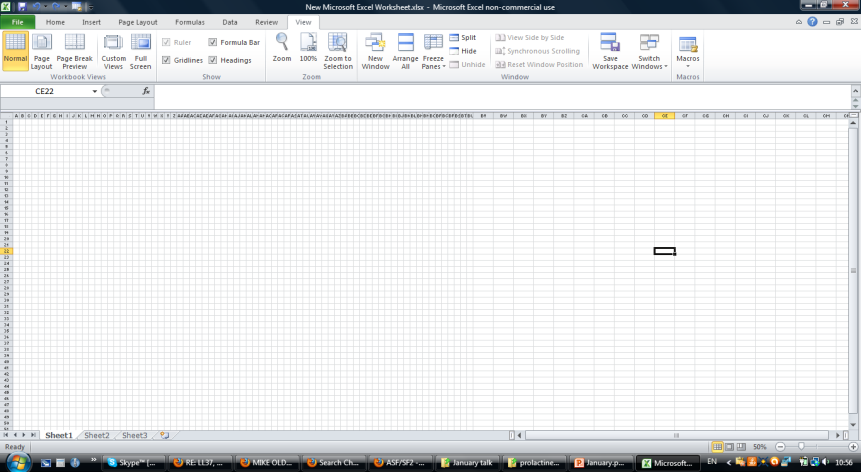


GGTTCAGCCATTGAGT

mRNA of gene #1

mRNA of gene #2

**B**


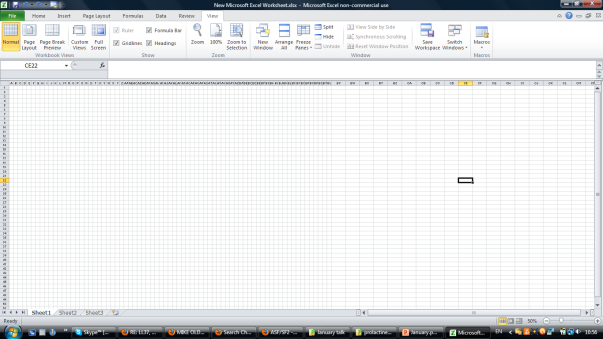

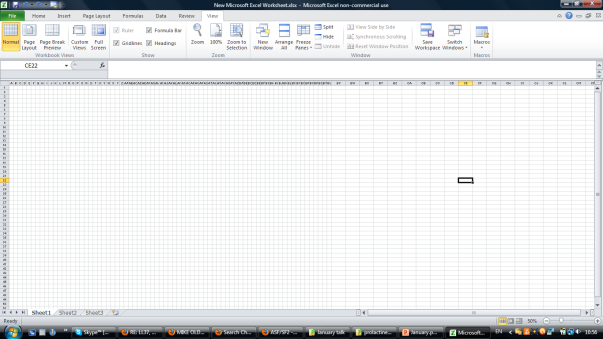

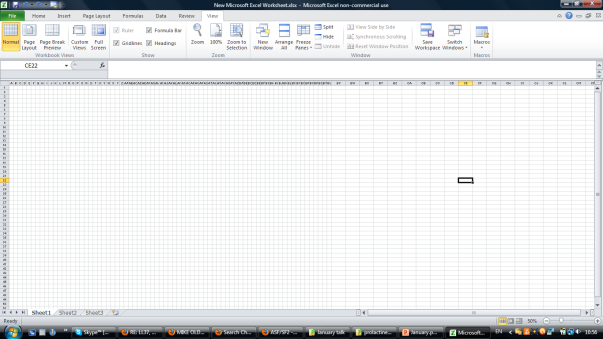

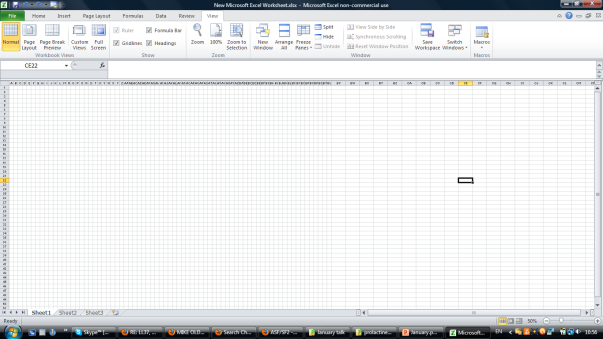

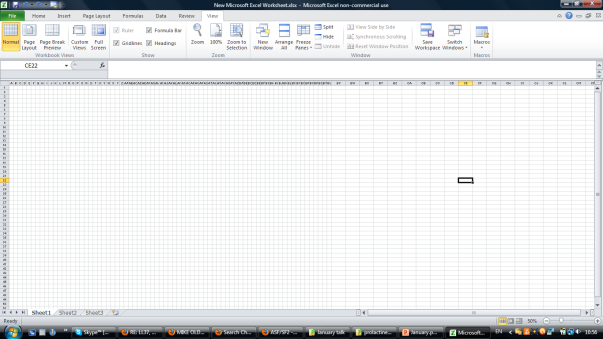

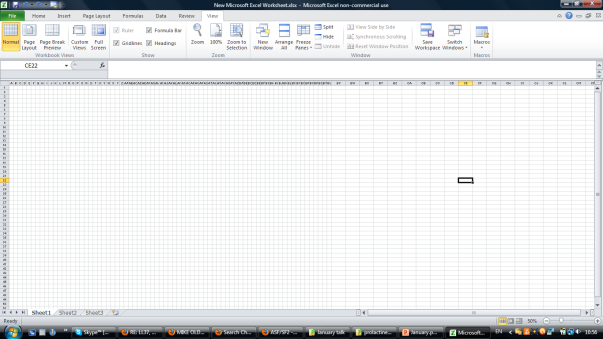

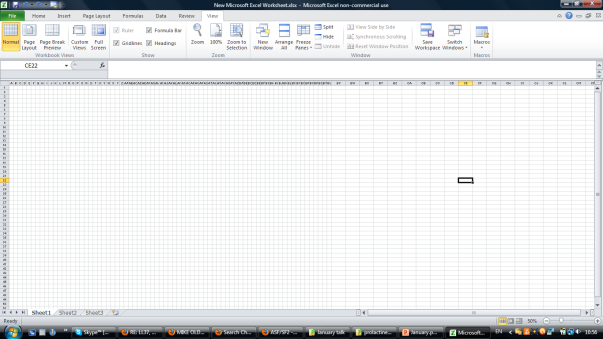

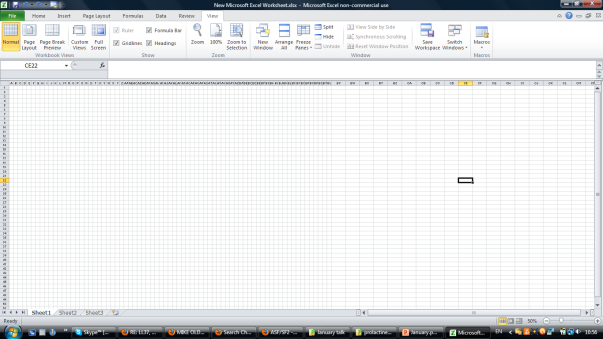

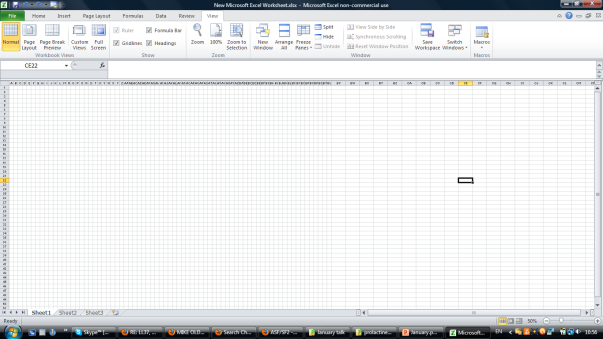


GGTTCAGCCATTGAGT
